# A Comparative Study of Ethylene Emanation upon Nitrogen Deficiency in Natural Accessions of *Arabidopsis thaliana*

**DOI:** 10.3389/fpls.2016.00070

**Published:** 2016-02-10

**Authors:** Hugues De Gernier, Jérôme De Pessemier, Jiajia Xu, Simona M. Cristescu, Dominique Van Der Straeten, Nathalie Verbruggen, Christian Hermans

**Affiliations:** ^1^Laboratory of Plant Physiology and Molecular Genetics, Interfacultary School of Bioengineers, Université Libre de BruxellesBrussels, Belgium; ^2^Trace Gas Research Group, Department of Molecular and Laser Physics, Institute for Molecules and Materials, Radboud UniversityNijmegen, Netherlands; ^3^Unit Hormone Signalling and Bio-Imaging, Laboratory of Functional Plant Biology, Department of Physiology, Ghent UniversityGhent, Belgium

**Keywords:** Arabidopsis, biomass, ethylene, natural variation, nitrogen

## Abstract

An original approach to develop sustainable agriculture with less nitrogen fertilizer inputs is to tackle the cross-talk between nitrogen nutrition and plant growth regulators. In particular the gaseous hormone, ethylene, is a prime target for that purpose. The variation of ethylene production in natural accessions of the model species *Arabidopsis thaliana* was explored in response to the nitrate supply. Ethylene was measured with a laser-based photoacoustic detector. First, experimental conditions were established with Columbia-0 (Col-0) accession, which was grown *in vitro* on horizontal plates across a range of five nitrate concentrations (0.5, 1, 2.5, 5, or 10 mM). The concentrations of 1 and 10 mM nitrate were retained for further characterization. Along with a decrease of total dry biomass and higher biomass allocation to the roots, the ethylene production was 50% more important at 1 mM than at 10 mM nitrate. The total transcript levels of *1-AMINOCYCLOPROPANE-1-CARBOXYLIC ACID SYNTHASES* (*ACS*) in roots and those of *ACC OXIDASES* (*ACO*) in shoots increased by 100% between the same treatments. This was mainly due to higher transcript levels of *ACS6* and of *ACO2* and *ACO4* respectively. The assumption was that during nitrogen deficiency, the greater biomass allocation in favor of the roots was controlled by ethylene being released in the shoots after conversion of ACC originating from the roots. Second, biomass and ethylene productions were measured in 20 additional accessions. Across all accessions, the total dry biomass and ethylene production were correlated negatively at 1 mM but positively at 10 mM nitrate. Furthermore, polymorphism was surveyed in ACC and ethylene biosynthesis genes and gene products among accessions. Very few substitutions modifying the amino acids properties in conserved motifs of the enzymes were found in the accessions. Natural variation of ethylene production could be further explored to improve Nitrogen Use Efficiency (NUE), in particular by manipulating features like the biomass production and the timing of senescence upon nitrogen limitation.

## Introduction

Plant growth requires profuse amount of nitrogen, since that element constitutes to nearby two percent of plant dry matter and is a component of key biological molecules like nucleic and amino acids, chlorophyll, and various metabolites. In agriculture, breeding strategies are urgently required to ameliorate Nitrogen Use Efficiency (NUE), in order to sustain the galloping world population growth and to preserve the environment from nitrogen (N) fertilizer overuse (Giles, 2005; Robertson and Vitousek, 2009; Godfray et al., 2010; Good and Beatty, 2011; Davidson et al., 2015). An innovative approach to enhance crop adaptability to nitrogen limitation is to tackle the cross-talk between mineral nutrition and biosynthetic pathways together with signaling cascades of plant growth regulators. Particularly the gaseous hormone, ethylene, is a prime target. Indeed, ethylene appears to regulate a wide range of morphological and developmental processes such as biomass production (Grichko and Glick, 2001; Khan, 2005), leaf cell expansion (Kieber et al., 1993; Rodrigues-Pousada et al., 1993), and lateral root formation (Swarup et al., 2007; Ivanchenko et al., 2008; Negi et al., 2008; Street et al., 2015). Moreover, ethylene is known to influence various physiological processes such as senescence timing (Jing et al., 2005; Ueda and Kusaba, 2015) and nutrient recycling (Nagarajan and Smith, 2012). The relevance of those biological processes in response to N availability is reviewed in this special issue (Khan et al., 2015). The depletion or excess of N on ethylene biosynthesis and signaling has already been examined by some authors (Mir et al., 2010; Iqbal et al., 2011, 2015). A transient and rapid increase of ethylene production was detected 1 h after transferring plants from low to high nitrate conditions (Tian et al., 2009) and reciprocally (Zheng et al., 2013). Effect of N deficiency on ethylene production has however not been characterized over a longer growth period yet. Besides, it is reported that ethylene modulates the expression of genes encoding major nitrate systems like *AtNITRATE TRANSPORTER 1.1 /NITRATE PEPTIDE TRANSPORTER FAMILY 6.3* (*NRT1.1*/*NPF6.3*) and *AtNRT2.1* (Tian et al., 2009; Zheng et al., 2013).

More generally, a large body of evidence indicates that ethylene is not solely associated with N but also with other mineral element availability (García et al., 2015; Thao et al., 2015; Song and Liu, 2015). For instance, ethylene production is elicited by the depletion of major essential elements like potassium (K) (Shin and Schachtman, 2004; Jung et al., 2009; Benlloch-Gonzàlez et al., 2010), phosphorus (P) (Borch et al., 1999), and magnesium (Mg) (Hermans et al., 2010b). The depletion of microelements such as iron (Fe) (Romera et al., 1999; Waters and Blevins, 2000; Romera and Alcántara, 2004; Molassiotis et al., 2005) and manganese (Mn) (Dorling et al., 2011) also enhances ethylene production. Likewise, the exposure to high concentrations of other essential trace elements like copper (Cu) (Arteca and Arteca, 2007) or non-essential ones like cadmium (Cd) (Rodríguez-Serrano et al., 2006; Arteca and Arteca, 2007; Schellingen et al., 2014) and selenium (Se) (Tamaoki et al., 2008) increases ethylene production in plants.

Enzymes in the ethylene biosynthetic pathway are crucial to modulate hormone production (Van de Poel and Van Der Straeten, 2014). Ethylene is synthesized from the amino acid methionine (Met) in successive steps, with the most limiting one being the transformation of the intermediate S-adenosyl-methionine (S-AdoMet) in 1-aminocyclopropane-1-carboxylic acid (ACC) by ACC synthases (ACS) (Yang and Hoffman, 1984; De Paepe and Van Der Straeten, 2005). Ethylene is finally produced from ACC through the action of ACC oxidases (ACO) with the reduction of oxygen and oxidation of a reducing agent, possibly ascorbate (Chae and Kieber, 2005; Lin et al., 2009). The Arabidopsis genome contains 12 *ACS* genes which encode eight functional ACC synthase proteins relatively similar in their polypeptidic sequences (Yamagami et al., 2003; Tsuchisaka and Theologis, 2004) and five *ACO* genes which belong to the 2-oxoglutarate dioxygenases family (Gómez-Lim et al., 1993; Raz and Ecker, 1999; Lin et al., 2009). The *ACS* and *ACO* genes are subjected to transcriptional and post-transcriptional regulations during plant development and stress conditions (Van Der Straeten et al., 2001; Wang et al., 2002; Tsuchisaka and Theologis, 2004; Lin et al., 2009; Yuan et al., 2010; Van de Poel and Van Der Straeten, 2014; Booker and DeLong, 2015). In Arabidopsis, mineral constraints are known to modulate the expression of *ACS* genes and mainly of *ACS2* and *ACS6.* For instance, up-regulation of *ACS* genes was observed in response to Mg deficiency (Hermans et al., 2010a,b), exposure to Cd (Schellingen et al., 2014) and Se (Van Hoewyk et al., 2008). However, reports about N supply effect on *ACS* gene expression are sparse and observations depend on the organ and developmental stage of the plants (Khan et al., 2015). Transferring plants from low to high nitrate conditions resulted in higher transcript levels of all *ACS* genes with the exception of *ACS9* (Tian et al., 2009). Resupplying nitrate to starved plants slightly increased *ACS6* transcript levels (Wang et al., 2002). Finally, the expression patterns and the functional redundancy of the ACO proteins are much less understood compared to ACS enzymes.

*Arabidopsis thaliana* is native to Europe and Central Asia and has a widespread geographical area, exposing it to various environmental selective pressures (Koornneef et al., 2004). Some examples of adaptive traits are the tolerance to drought, salinity, and frost (Rus et al., 2006; Bouchabke et al., 2008; McKhann et al., 2008; Des Marais et al., 2012; Kesari et al., 2012; Wollenberg and Amasino, 2012). The natural variation offered by that species permitted to gain insights into adaptive metabolic and morphological strategies to low N conditions (Loudet et al., 2003; Chardon et al., 2010, 2012; De Pessemier et al., 2013).

Arabidopsis is a model species with small genome but a weed without any agronomic value. However, it is closely related to Brassica crops having complex genomes, resulting from multiple rounds of polyploidy in their ancestry (Trick et al., 2009). In that context, the biodiversity offered by Arabidopsis could help deciphering mechanisms controlling plant developmental processes dependent on ethylene. This can be important for improving NUE in Brassica crops (Mir et al., 2010; Iqbal et al., 2011).

This study describes an *in vitro* approach to screen for variation in the ethylene emanation in response to the nitrate supply in a core set of Arabidopsis natural populations with diverse geographic origins. The experiment setup was first tested on Columbia-0 (Col-0) and then applied to other accessions. Furthermore, Col-0 accession was subjected to detailed physiological and molecular investigations in order to characterize the interaction between nitrate supply and ethylene production. How such research can help the breeding of future high-NUE cultivars is further discussed.

## Materials and methods

### *In vitro* culture

Seeds of *A. thaliana* accessions were obtained from the *A. thaliana* Resource Centre for Genomics, INRA, Versailles, France. A panel of 24 accessions that maximizes the genetic diversity of the species (McKhann et al., 2004) and the reference Columbia-0 (Col-0) accession was composed. The information collected on the accessions used in this study is presented in Table S1. Batches of seeds simultaneously generated from the original stocks were used for *in vitro* growth experiments. The following four accessions were discarded due to poor germination rate or insufficient number of seeds for carrying experiments: Alcalá de Hernares (Alc-0), Canary Island-0 (Can-0), Greenville-0 (Gre-0), and Sakata. The following 20 accessions and Col-0 were retained for further phenotyping procedure: Akita, Bologna-1 (Bl-1), Bulhary-1 (Blh-1), Burren-0 (Bur-0), Catania-1 (Ct-1), Cape Verde Islands-0 (Cvi-0), Edinburgh-0 (Edi-0), Geneva-0 (Ge-0), Ibel Tazekka-0 (Ita-0), St Jean Cap Ferrat (JEA), Kaunas-0 (Kn-0), Mulhen-1 (Mh-1), Martuba-0 (Mt-0), Konchezero (N13), Oystese-0 (Oy-0), le Pyla-1 (Pyl-1), Shahdara River (Sha), Stockholm-0 (St-0), Stobowa-0 (Stw-0), and Tsu-0. Seeds were sterilized with ethanol 70% (v/v) for 10 min and hypochlorite 20% (v/v) solution for 5 min. Hundred seeds were plated on 1x Murashige and Skoog medium modified with nitrate as the sole N source, 1% sucrose, 0.6% agar, and pH = 5.7 (Hermans et al., 2010c). In the initial screen with Col-0 accession, nitrate concentrations were 0.5, 1, 2.5, or 10 mM (fully supplied condition). The nitrate concentrations of 1 and 10 mM were retained for screening 20 additional Arabidopsis accessions. In order to avoid inducing K depletion in media with lower nitrate concentrations (< 10 mM), KCl salt was added as a replacement for KNO_3_, as described in De Pessemier et al. (2013). Seeds were stratified at 4°C for two days in darkness, and the plates were horizontally incubated in a culture chamber at a temperature of 22°C and a constant light regime of 75 μmol photons m^−2^ s^−1^. Thirteen days after germination, physiological parameters and ethylene production of the seedlings were measured.

### Nitrogen and carbon determination

Sample dry weights of 10–50 mg were analyzed with a vario MAX cube (Elementar, Germany) for simultaneous C and N determination at Centre pour l'Agronomie et l'Agro-Industrie de la province de Hainaut, Belgium (CARAH).

### Pigments determination

Pigments were extracted from frozen shoot organs (~20 mg fresh weight) according to the procedures described in Misyura et al. (2013) and Teng et al. (2005).

### Ethylene production measurement

Headspace samples were analyzed with a laser-based photoacoustic ethylene detector (ETD-300, Sensor-Sense, Nijmegen, the Netherlands). A valve control box allowed automated sampling of ethylene production under a stop-and-flow routine as described in Hermans et al. (2011) (Figure S1). Ethylene production of six Petri dishes was measured sequentially. Gas accumulated during 1 h and then was flushed to the detector during 12 min. Each sample was measured during at least two sequences and the average ethylene production was calculated. Values were corrected by the signal recorded for an empty agar plate. The experiment was run in at least four replicates for each accession and nitrate concentration.

### Gene expression analysis

Total RNA was extracted from shoot and root tissues seedlings grown for 13 days after germination, using the Maxwell LEV Plant RNA kit with the Maxwell 16 Research Instrument (Promega Fitchburg). The first-strand cDNA was synthesized using Promega GoScript Reverse Transcription System. Quantitative PCR analyses were carried with the Takyon qPCR Kit (Eurogentec) using the PikoReal™ Real-Time PCR System (Thermo Scientific). Gene-specific forward and reverse primers are listed in Table S2. PikoReal™ software was used for analyzing and quantifying qPCR curves. The two stably expressed reference genes *ACTINE 2* (*ACT2*) and *POLYUBIQUITIN 10* (*UBQ10*) were used for the normalization of all target gene expression.

### Genomic analysis and association tests

Genomic sequences were retrieved from the 250k SNP data published by Atwell et al. (2010) and Li et al. (2010) for Alc-0, Blh-1, Bur-0, Can-0, Ct-1, Cvi-0, Edi-0, Ge-0, Jea, Kn-0, Mh-1, Mt-0, N13, Oy-0, Sha, St-0, Stw-0 and Tsu-0, and from the Salk Arabidopsis 1001 Genomes database (http://signal.salk.edu/atg1001/3.0/gebrowser.php) for Bl-1, Gre-0, and Sakata. Sequences were missing for Akita, Ita-0 and Pyl-1. TAIR10 gene models were plotted with the R package *genoPlotR* (Guy et al., 2010). The density of single nucleotide polymorphisms (SNPs) in *ACS* and *ACO* genes was defined by the SNP number divided by the length of the corresponding exon. Association tests between SNPs (retrieved from 250k SNP data set) and ethylene production were performed with GEMMA (Zhou and Stephens, 2014).

### Statistical treatment

All statistical analyses were conducted using the R software (R Core Team, 2014). Mean comparisons were performed by analysis of variance (ANOVA). For association tests between paired variables (phenotype traits or SNPs), Pearson's product moment correlation coefficient (r) was used. Linear regression models were subsequently designed when needed. Adequate model fits were ensured through residual investigation with the help of the diagnostic plots implemented in the software.

## Results

### Influence of nitrate supply on biomass production, nitrogen, carbon, and pigment concentrations in columbia-0 accession

**Figure 1:**
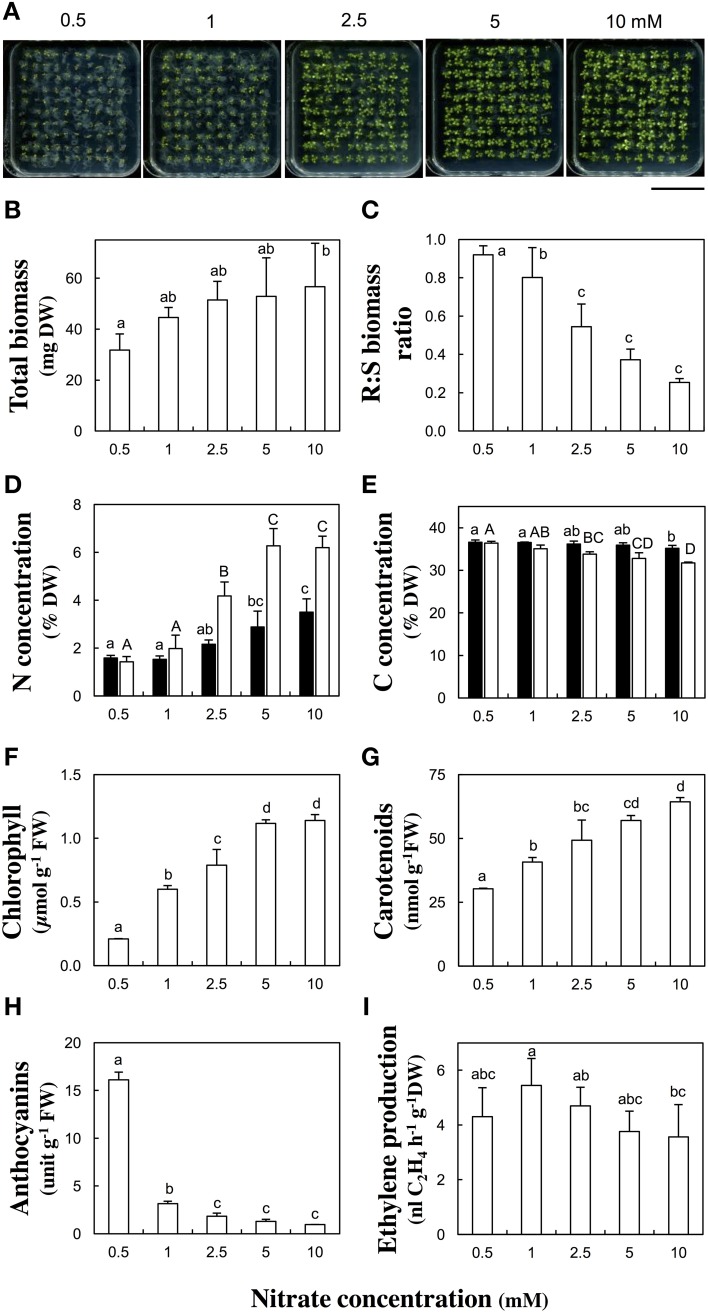
**Biomass production, nitrogen and carbon tissue concentration, pigment levels, and ethylene emanation in response to nitrate supply in Columbia-0 accession**. *Arabidopsis thaliana* Col-0 seedlings were grown across a range of nitrate concentrations (0.5, 1, 2.5, 5, or 10 mM) and harvested 13 days after germination. **(A)** Representative pictures of petri plates containing 100 seedlings. Scale bar: 5 cm, **(B)** Total dry biomass, **(C)** Root to shoot dry biomass ratio, **(D,E)** Total nitrogen **(D)** and carbon **(E)** concentrations per dry weight of root (black columns) or shoot (white columns) tissues. **(F–H)** Pigment concentrations per fresh weight of shoot tissues: total chlorophyll (Chl*a*+*b*) **(F)**, carotenoids **(G)** and anthocyanins **(H)**, **(I)** Ethylene production per hour and per dry weight of total seedlings. Mean of four samples (100 pooled seedlings) ± SD. Letters indicate significant differences (Tukey's HSD test, *p* < 0.1).

Experimental conditions were first established with the reference Col-0 accession, which was challenged to a range of five nitrate concentrations (0.5, 1, 2.5, 5, or 10 mM) during *in vitro* culture for 13 days after germination (Figure 1). The total dry biomass of hundred pooled seedlings was slightly different [*F*_(5, 18)_ = 2.24, *p* = 0.095] between nitrate treatments. It gradually increased with the nitrate supply rising and was almost double between 0.5 mM and 10 mM nitrate (Figure 1B). The nitrate availability significantly [*F*_(5, 16)_ = 33.63, *p* < 0.001] affected the root to shoot dry biomass ratio. The ratio decreased from 0.91 to 0.25 with the nitrate supply augmenting (Figure 1C). Furthermore, N and carbon (C) tissue concentrations were measured in root and shoot organs. The N concentrations rose with increasing nitrate supply (Figure 1D). They were up to four times lower in shoots and two times lower in roots at 0.5 mM compared to 10 mM nitrate. Such important differences in C concentrations were not observed between the nitrate treatments. The C concentrations slightly decreased with increasing nitrate supply (Figure 1E). The difference of C concentrations was more prononced in shoots (−15%) than in roots between 0.5 and 10 mM nitrate supplies.

Because the leaves of seedlings grown at low nitrate supply (< 1 mM) showed chloroses and purple shades (Figure 1A), the chlorophyll, carotenoid, and anthocyanin concentrations were quantified, together with a leaf senescence molecular marker. The total chlorophyll concentrations severely decreased at concentrations lower than 2.5 mM nitrate (Figure 1F). Chlorophyll concentrations were five or two times lower in seedlings grown respectively at 0.5 or 1 than at 10 mM nitrate. Along with a reduction in chlorophyll concentration, the expression of *SENESCENCE-ASSOCIATED GENE 12* (*SAG12*) was massively triggered in N-deficient shoot tissues (Table S3). Carotenoid concentrations decreased at low nitrate supplies (Figure 1G). A decrease of one half or one third of their level was observed respectively at 0.5 or 1 mM compared to 10 mM nitrate. Anthocyanin concentrations increased by a factor 16 in seedlings grown at 0.5 mM and by a factor three at 1 mM compared to 10 mM nitrate (Figure 1H).

### Influence of nitrate supply on ethylene production and expression of genes in the ethylene biosynthetic pathway in columbia-0 accession

**Figure 2:**
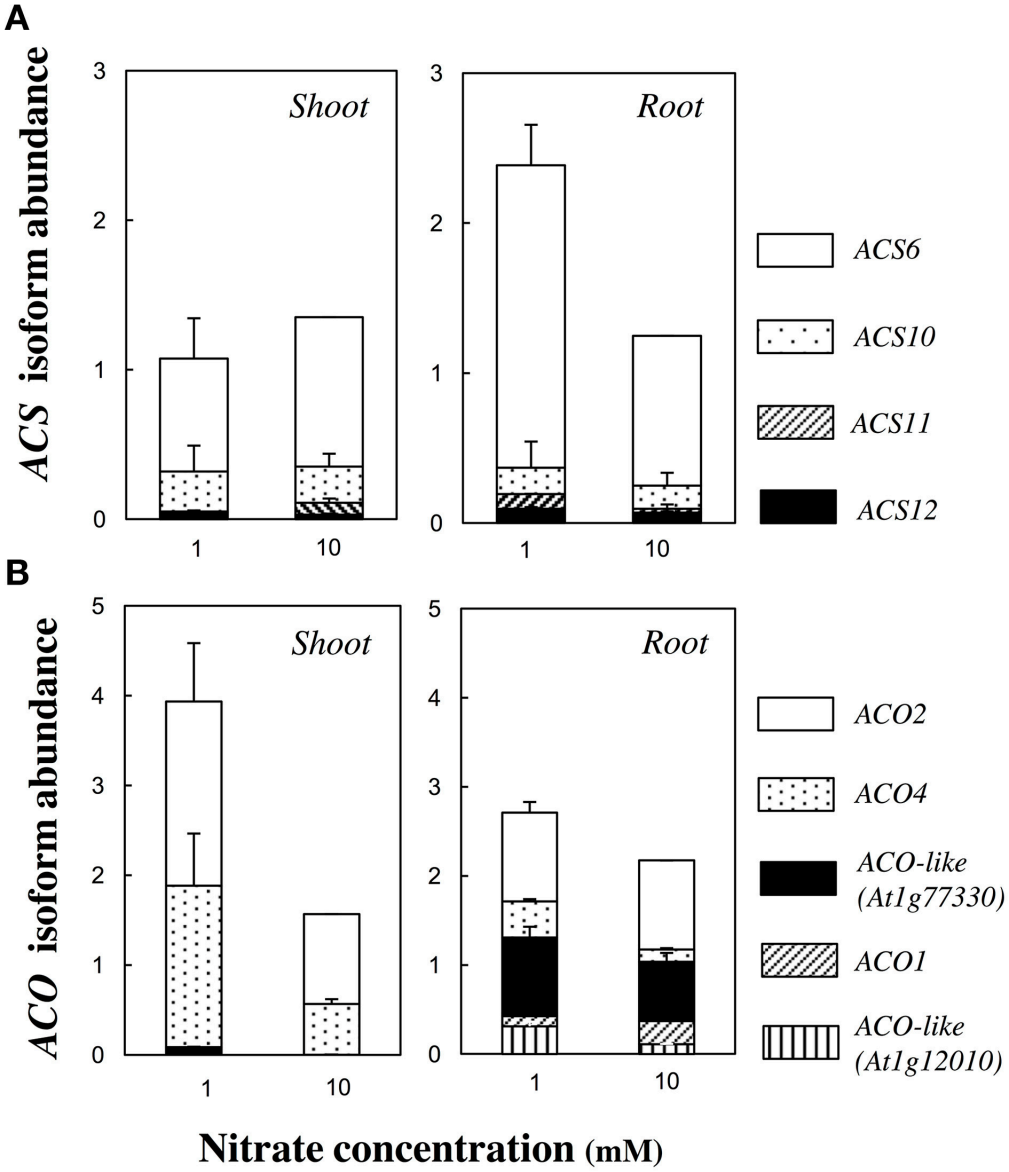
**Transcript abundance of *ACC SYNTHASE* and *ACC OXIDASE* multigene family**. Transcript levels of the most highly expressed *ACS* **(A)** and *ACO* **(B)** members are presented in root and shoot tissues of Col-0 seedlings grown at 1 or 10 mM nitrate, 13 days after germination. Data represent mean abundance with the abundance of the most highly expressed (normalized by *ACT2* and *UBQ10* levels) family member in one organ, set as one under 10 mM nitrate condition (*ACS6* = 1 and *ACO2* = 1, respectively for *ACS* and *ACO* families). Mean of two or three pools of 100 organs ± SD (each sample assessed by three technical replicates).

The ethylene production was measured 13 days after germination and normalized by the total dry biomass. The ethylene production was low at 0.5 mM, reached its highest value at 1 mM and then slowly decreased with the nitrate concentration increasing (Figure 1I). However, only one significant [*p* < 0.1, Tukey's HSD test] difference was found between 1 and 10 mM nitrate treatments. Therefore, those two nitrate concentrations were retained for screening the other accessions (see below). That bell-shaped response was also observed upon normalization of the ethylene production by seedling number. The transcript abundance of *ACC SYNTHASES* and *ACC OXYDASES* multigene families was monitored in plant organs. The relative contribution of the most highly expressed genes of each family is shown in Figure 2 and the quantification of all isoform transcripts in Table S3. The total *ACS* transcript abundance in roots at 1 mM was twice as high as at 10 mM nitrate (Figure 2A). That increase was due in a large proportion to the induction of *ACS6* transcript levels, and in a lesser proportion to the induction of *ACS10, ACS11*, and *ASC12*. By contrast, the total *ACS* transcript abundance in shoots was relatively unchanged (Figure 2A). The total *ACO* transcript abundance in shoots at 1 mM was twice as high as at 10 mM nitrate (Figure 2B). Induction of transcript levels of *ACO2* and *ACO4* predominantly contributed to the total *ACO* expression level increase. In roots, all *ACO* isoforms were highly expressed but generally contributed to a slight increase of the total *ACO* expression level at 1 mM compared to 10 mM nitrate (Figure 2B).

### Influence of nitrate supply on biomass and ethylene production in a core set of arabidopsis accessions

**Figure 3:**
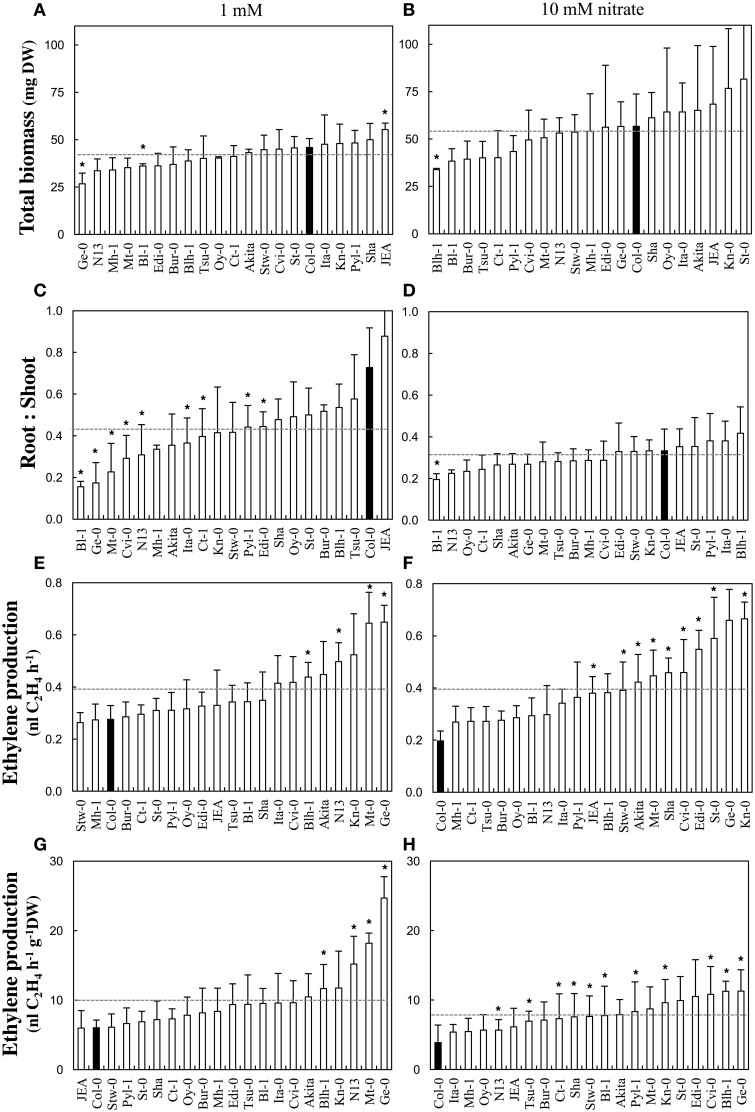
**Biomass production and ethylene emanation in response to nitrate supply in Arabidopsis accessions**. Accessions were grown at 1 or 10 mM nitrate supplies and harvested 13 days after germination. **(A,B)** Total dry biomass, **(C,D)** Root to shoot dry biomass ratio, **(E,F)** Ethylene emanated per hour and per hundred seedlings, **(G,H)** Ethylene emanated per hour and per dry weight. Parameters were measured at 1 mM **(A,C,E,G)** and 10 mM **(B,D,F,H)** nitrate. Mean of four samples (100 pooled seedlings) ± SD. Asterisks represent significant (*p* < 0.1, Kolmogorov-Smirnov two-sided test) differences between one accession and Col-0.

Biomass parameters and ethylene production were measured in 20 additional accessions (Akita, Bl-1, Blh-1, Bur-0, Ct-1, Cvi-0, Edi-0, Ge-0, Ita-0, JEA, Kn-0, Mh-1, Mt-0, N13, Oy-0, Pyl-1, Sha, St-0, Stw-0, and Tsu-0) grown in the same conditions as described above (Figure 3). An analysis of variance was conducted for each measured variable in order to investigate individual effects of treatment (nitrate supply) and genotype (accession) as well as their interaction *treatment* × *genotype*. Across all accessions, the total dry biomass normalized to one-hundred seedlings was significantly [*F*_(1, 113)_ = 1.84, *p* < 0.001] lower at 1 mM (42.1 ± 9.3 mg) than at 10 mM (54.4 ± 22.8 mg) (Figures 3A,B). Moreover, the total dry biomasses were significantly [*F*_(20, 113)_ = 1.84, *p* = 0.024] different between accessions regardless of the nitrate supply. Finally, there was no marked biomass changes in response to nitrate supply between the accessions [no significant *treatment* × *genotype* effect: *F*_(20, 113)_ = 0.927, *p* = 0.555]. The biomasses were invariably lower at 1 mM than at 10 mM. As expected, most accessions significantly [*F*_(1, 113)_ = 41.552, *p* < 0.001] allocated more biomass to the roots at 1 mM (root to shoot dry biomass ratio = 0.47 ± 0.22) than at 10 mM (0.3 ± 0.09) but some accessions did not have that behavior [significant *treatment* × *genotype* effect: *F*_(20, 113)_ = 2.829, *p* < 0.001] (Figures 3C,D). Strikingly distinct nitrate-response occurred between accessions while considering the amounts of ethylene emanated per hour and per hundred seedlings [significant *treatment* × *genotype* effect: *F*_(20, 113)_ = 1.778, *p* = 0.031]. Nevertheless, on the average of all accessions, the ethylene production did not significantly [*F*_(1, 113)_ = 0.584, *p* = 0.447] differ between the nitrate treatments (0.39 ± 0.13 vs. 0.39 ± 0.18 nl C_2_H_4_ h^−1^ respectively at 1 and 10 mM nitrate; Figures 3E,F). Consequently, accessions with low biomass emanated similar amounts of ethylene per hour than those with high biomass, so that the ethylene production normalized by the total dry biomass was significantly [*F*_(1, 113)_ = 14.706, *p* < 0.001] 1.3 times higher at 1 mM than at 10 mM nitrate (10 ± 5.3 vs. 7.8 ± 3.7 nl C_2_H_4_ h^−1^ g^−1^ DW respectively; Figures 3G,H). Thirteen accessions showed such behavior in response to nitrate supply (Ita-0, Mt-0, N13, and Ge-0 presenting the largest differences between1 mM and 10 mM) and the other ones displayed an opposite trend (St-0, Pyl-1, Stw-0, Cvi-0, Edi-0, Sha, JEA, and Ct-1, ordered by decreasing differences).

**Figure 4:**
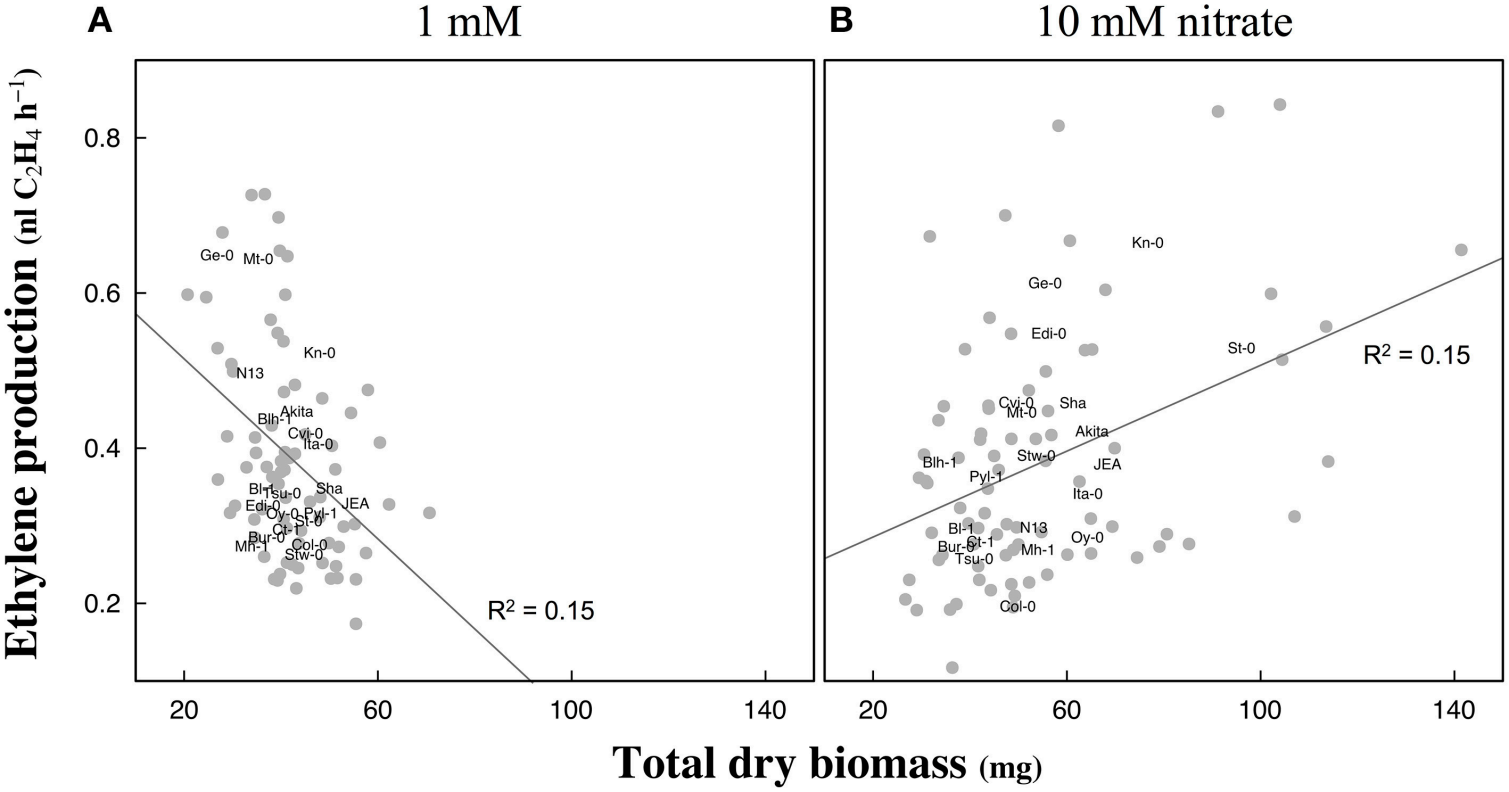
**Correlation between biomass production and ethylene emanation in response to nitrate availability in Arabidopsis accessions**. Hundred seedlings of each accession were grown at 1 mM **(A)** or 10 mM **(B)** nitrate supplies and harvested 13 days after germination. Total dry weight biomass and ethylene emanation normalized to hundred seedlings were measured in four replicates. Accession names indicate means of four replicates. Linear regression models indicated a significant negative association between the total dry biomass and ethylene emanation at 1 mM and a positive association at 10 mM (slopes different from zero: *p* < 0.001). Model details at 1 mM: *p* < 0.001, adjusted *R*^2^ = 0.15, *F*-statistic = 14.01 on 1 DF, residual SE = 0.124 on 72 DF; and 10 mM: *p* < 0.001, adjusted *R*^2^ = 0.15, *F*-statistic = 14.82 on 1 DF, residual SE = 0.146 on 76 DF.

The likely associations between the amount of ethylene emanated per hour and total dry biomass were investigated for both nitrate supplies. The linear regression models revealed a significant [*R*^2^ = 0.15, *p* < 0.001] negative association between the two parameters at 1 mM but a significant [*R*^2^ = 0.15, *p* < 0.001] positive association at 10 mM nitrate (Figure 4).

### Influence of nitrate supply on pigment concentration in a core set of arabidopsis accessions

Pigment concentrations were measured in accessions grown at 1 or 10 mM nitrate (Table S4). For the three pigment classes, highly significant [*p* < 0.001] treatment, genotype and *treatment* × *genotype* interaction effects were found. Across all accessions, the chlorophyll *a*+*b* concentration was two times lower at 1 mM (0.54 ± 0.12 μmol g^−1^ FW) than at 10 mM (1.06 ± 0.21 μmol g^−1^ FW) nitrate treatment [*F*_(1, 80)_ = 3509.46, *p* < 0.001]. Only Cvi-0 showed a slightly higher chlorophyll concentration at 1 mM than at 10 mM. The responses of the accessions to nitrate supply were significantly different [*treatment* × *genotype* interaction: *F*_(19, 80)_ = 33.05, *p* < 0.001]. In the same way, the carotenoid concentration at 1 mM (46.75 ± 5.95 nmol g^−1^ FW) was 1.3 times lower at 1 mM than at 10 mM [61.53 ± 12.31 nmol g^−1^ FW; *F*_(1, 80)_ = 491.49, *p* < 0.001]. The *treatment* × *genotype* effect was also significant [*F*_(19, 80)_ = 16.99, *p* < 0.001] and five accessions (Cvi-0, N13, Akita and Blh-1, Mh-1) showed either higher carotenoid concentrations at 1 mM than at 10 mM, either no difference between treatments. Finally, the anthocyanin concentration was 12 times more important at 1 mM (13.8 unit g^−1^ FW) than at 10 mM nitrate [1.18 unit g^−1^ FW; *F*_(1, 80)_ = 4951.34, *p* < 0.001]. The *treatment* × *genotype* effect was significant [*F*_(19, 80)_ = 35.56, p < 0.001] and Cvi-0 presented the lowest difference between the two nitrate treatments among the accession panel.

Eventually, the average pigment concentrations were computed for each treatment-genotype pair and combined with the corresponding average ethylene productions (normalized by fresh weight). Correlation tests were conducted after a paired resampling procedure was applied to increase the number of replicates (associated methods and results are in Table S5). They revealed a negative correlation between chlorophyll *a*+*b* concentration and ethylene production, and a positive correlation between anthocyanin concentration and ethylene production for both nitrate treatments. Finally, the carotenoid concentration was positively correlated with the ethylene production at 1 mM but negatively at 10 mM nitrate (Table S5).

### Polymorphism analysis of enzymes in the ethylene biosynthetic pathway

**Figure 5:**
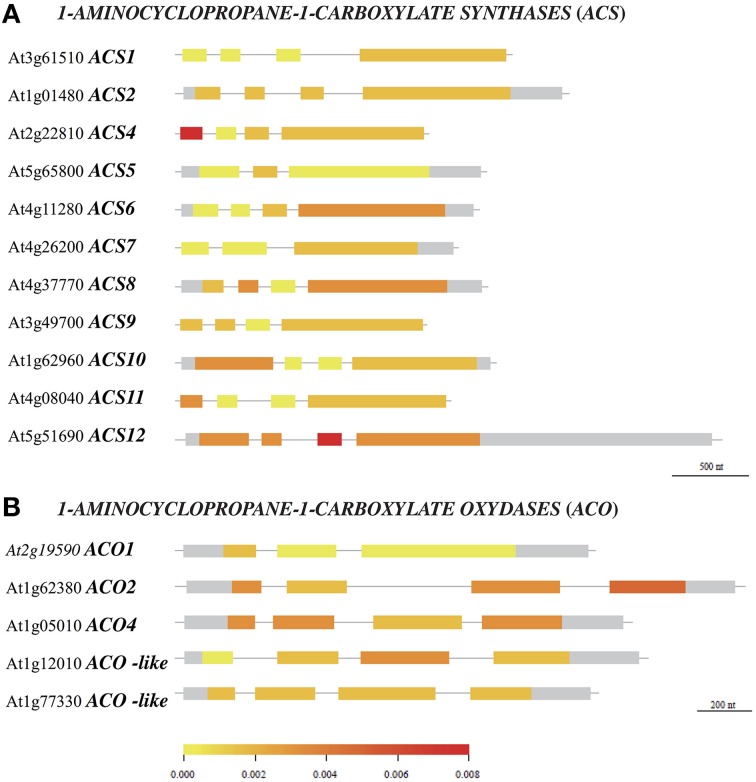
**Polymorphism density in DNA coding sequences of *ACC SYNTHASES* and *ACC OXYDASES* genes**. Aivailable coding gene sequences of *ACS* **(A)** and *ACO* **(B)** for 21 accessions from the McKhann et al. (2004) core set were compared to the corresponding Col-0 sequences retrieved from 250k SNP data published in Atwell et al. (2010) and Li et al. (2010). The density of single nucleotide polymorphism, defined as the number of SNPs per base pair in a given exon, is indicated by the color scale.

**Figure 6:**
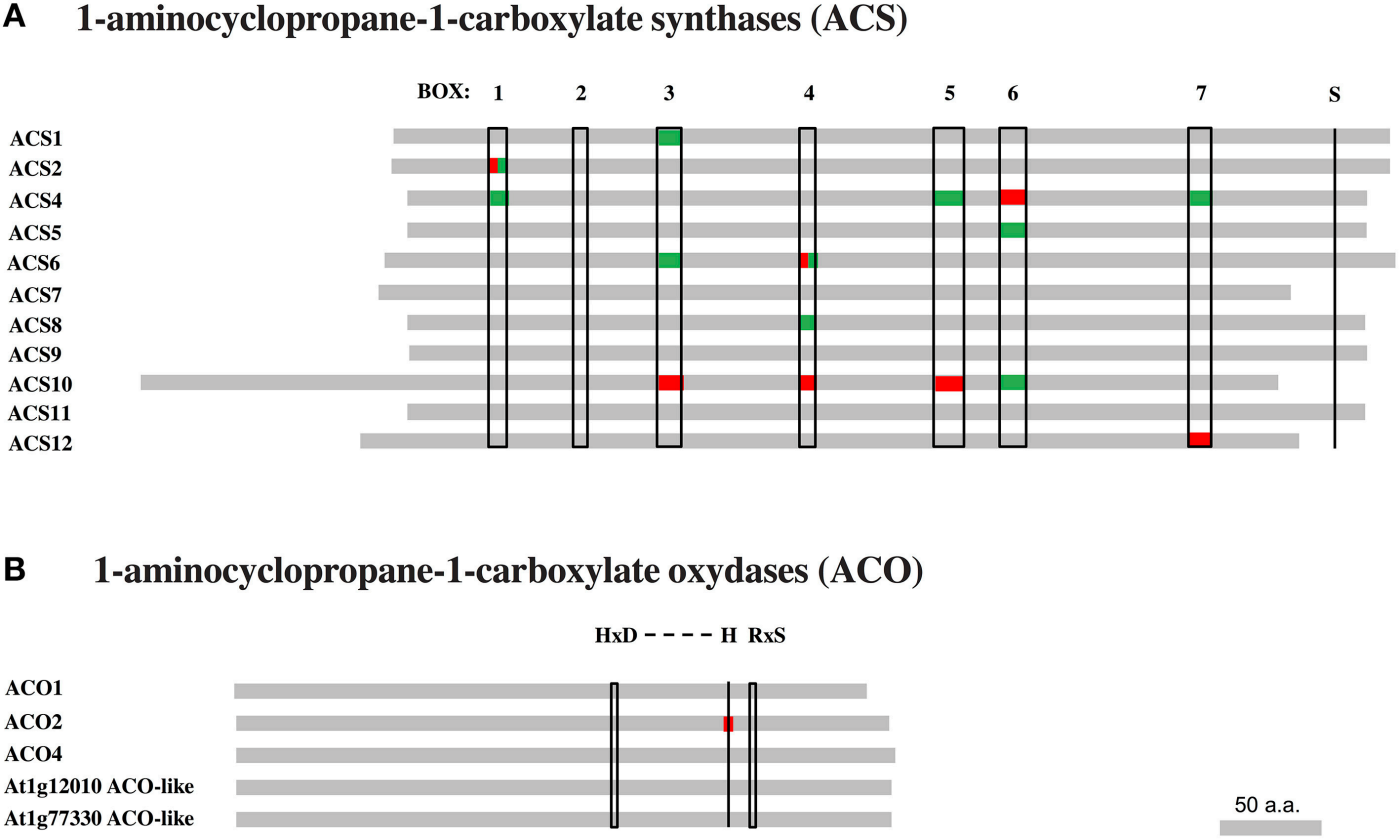
**Amino acid sequence alignment of ACC synthase and ACO oxydase proteins**. Available protein sequences of 21 accessions from the McKhann et al. (2004) core set were compared to the corresponding Col-0 sequences retrieved from 250k SNP data published in Atwell et al. (2010) and Li et al. (2010). Amino acid substitutions are indicated for conserved domains and motifs of ACS and ACO protein families. **(A)** The sequences of 11 ACS isozymes present seven conserved domains marked as boxes 1–7. The S residue marked at the sequence end of ACS1-6, 8, 9, and 11 is a putative phosphorylation site. **(B)** In ACO proteins, the so-called “2-His-1-carboylate facial triad” (HxD…H) motif is involved in co-factor (Fe^2+^) binding pocket, while the RxS motif is critical for substrate (ascorbate) binding pocket. The presence of one or more synonymous (green) or non-synonymous (red) amino acid substitutions in a given item is indicated.

Polymorphism variation present in *ACS* and *ACO* genes and proteins was compared along 21 accessions from the original McKhann et al. (2004) diversity panel. That initial survey was meant to highlight some naturally-occurring genetic differences which could be responsible for ethylene production differences observed between genotypes. First, available DNA coding sequences were compared to the corresponding Col-0 sequences and the SNP density was calculated in a given exon (Figure 5). The survey identified the first exon of *ACS4* and third one of *ACS12* with the highest SNP densities (Figure 5A). Overall, *ACS6* and *ACS12* were the most polymorphic genes with height SNPs (total exon length 1753 bp) and 17 SNPs (total exon length 3179 bp) respectively among *ACS* genes and *ACO4* with five SNPs (total exon length 1364 bp; Table S6). Second, particular attention was given to nucleotide substitutions leading to the same amino acid (synonymous or neutral) or resulting in amino acid variants (non-synonymous) in protein sequences (Figure 6, Figures S2, S3). Furthermore, non-synonymous substitutions were distinguished between conservative and non-conservative ones, if they respectively maintained or modified the amino acid property. In ACS proteins, seven conserved boxes and one phosphorylation site (Yamagami et al., 2003) were closely surveyed (Figure 6A). Within those boxes, non-conservative substitutions were found in a rather small number of accessions: in box 1 of ACS2 for Can-0, Edi and Kn-0, box 6 of ACS4 for Bur-0, box 4 of ACS6 for Ge-0, box 3 of ACS10 for Can-0, box 4 of ACS10 for Edi-0, Oy-0 and Sha, box 5 of ACS10 for all accessions and box 7 of ACS12 for Can-0, Cvi-0, Ge-0, Mt-0, St-0, and Stw-0 (Figure S2). In addition, only one of the 11 invariant amino acids (Yamagami et al., 2003) among ACS isozymes found synonymous substitution in ACS8 for Alc-0, Blh-1, Edi-0, and Sakata (Figure S2). No substitution originated from the putative phosphorylation site at the end of ACS1, 6, 8, 9, and 11 sequences. In ACO proteins, two conserved motifs involved in co-factor (Fe^2+^) and co-substrate (ascorbate) binding pockets (Seo et al., 2004; Yuan et al., 2010) were surveyed (Figure 6B). Only one non-conservative substitution was observed in the co-factor binding pocket in ACO2 sequence of Alc-0 (Figure S3).

## Discussion

Mineral nutrient constraints can affect hormonal level and signaling, and in turn, hormones can alter mineral homeostasis and biomass production. This study illustrated such intricate interplay between biomass production, biomass allocation to organs, and ethylene production in response to nitrate supply.

### Columbia-0 response to low nitrate supply

Our morphological observations on Col-0 accession during N deficiency were consistent with previous reports (Hermans et al., 2006, 2010c, 2011; De Pessemier et al., 2013). Low nitrate supply reduced biomass production and increased the root to shoot biomass ratio (Figures 1B,C). Higher transcript levels of *ACS6* in roots and of *ACO2* and *ACO4* in shoots (Figure 2) were mirrored by higher ethylene production (Figure 1I) in N-deficient seedlings. That tissue-specific transcription increase of genes encoding the ACC synthesizing vs. ethylene synthesizing enzymes, may indicate that ACC accumulated in roots in response to low nitrate and was converted to ethylene by ACO in roots. Long distance ACC transport was previously observed upon hypoxia and salinity exposure (reviewed in Van de Poel and Van Der Straeten, 2014). Moreover, Lynch and Brown (1997) speculated that ethylene may play an important role in mediating plasticity of plant responses to nutrient stress, especially root responses to P deficiency. Our data indicated that the nitrate supply largely modulated ethylene biosynthesis with a strong influence on plant morphology and biomass allocation (Figures 1, 4). The increase in biomass allocation to the roots at the expense of the shoots during N deficiency might be controlled by ethylene, released in the shoot upon conversion of ACC originating from roots. In Arabidopsis, ethylene inhibits leaf expansion (Van de Poel et al., 2015). Ethylene could therefore steer the alteration in root-shoot biomass ratio, with restriction of shoot expansion when N supply is low. However, that effect is probably primarily controlled by auxin or cytokinin, known to modulate ethylene biosynthesis and signaling, particularly at higher concentrations (Van de Poel et al., 2015). Thus, the alteration of biomass allocation between organs might be fine-tuned by ethylene as part of an intricate network in cross talk with other hormones (Khan et al., 2015).

Our physiological observations may reflect an ethylene-mediated senescence program (Qiu et al., 2015). Decreased chlorophyll concentrations (Figure 1F) and high transcript levels of *SAG12* (Figure S3) in N-deficient seedlings are obvious symptoms of senescence (Lohman et al., 1994; Qiu et al., 2015). Such severe yellowing was probably due to chloroplast degradation and nutrient recycling (Ueda and Kusaba, 2015). Low carotenoid concentration (Figure 1G) indicated that the color of N-deficient shoots was due to unmasking of yellow pigments rather than higher synthesis. Finally, the high anthocyanin concentration (Figure 1H) was consistent with earlier observations during N deficiency (Peng et al., 2008) and could reflect the activation of the phenylpropanoid pathway possibly by ethylene, as suggested by Khan et al. (2015). It is noteworthy that P deficiency can also cause anthocyanin accumulation but in that case, ethylene signaling plays a negative regulatory role in anthocyanin biosynthesis (Lei et al., 2011). To determine if ethylene action actually triggers chlorophyll degradation and/or anthocyanin production, ethylene signaling or biosynthesis mutants must be challenged to low N supply. That question urges further research in the area.

### Genetic variation exists for ethylene production in arabidopsis natural accessions

A core set of Arabidopsis accessions was screened for the ethylene production. Up to now, the assessment of natural variability for that trait is very limited. A two-fold variation of ethylene production upon exposure to abiotic stresses is reported between some foremost studied Arabidopsis accessions: Col-0, Landsberg-0 (Ler-0), and Wassilewskija-2 (Ws-2) (Tamaoki et al., 2008; van Zanten et al., 2010). Here, a six-fold variation of ethylene production was observed between JEA and Ge-0, which were positioned at opposite ends under low nitrate supply (Figure 3G). This clearly illustrates that natural genetic variation does exist for ethylene production within the *A. thaliana* species. Polymorphism in the ethylene biosynthetic enzymes could be responsible for phenotypic differences observed between Arabidopsis accessions. Our analysis showed that the genomic sequences of *ACS6, ACS12*, and *ACO4* have higher divergence than any other family members (Figure 5, Table S6). It is noteworthy that *ACS6* and *ACO4* are the most up-regulated genes during low nitrate treatment in Col-0 genotype (Figure 2). Despite the limited number of accessions analyzed in the present study, an association test was performed between the retrieved genomic sequences of *ACS* and *ACO* genes (Figure 5) and ethylene production (Figure 3). Only *ACS4* showed significant association [*p* < 0.001] with ethylene production measured in 17 accessions grown at 10 mM nitrate (Table S7). It is worth mentioning that the statistical power of the analysis is low and the false discovery rate high.

Non-synonymous substitutions of amino acids can potentially change the protein function, although very little divergence was found in catalytic and phosphorylation sites of the ACC and ethylene biosynthetic enzymes (Figure 6). Though, a nucleotide substitution that does not modify the amino acid sequence, can yet affect the translation efficiency, disrupt splicing and modify protein structure, abundance and even substrate specificity (Kimchi-Sarfaty et al., 2007; Parmley and Hurst, 2007; Kesari et al., 2012). Our study only focused on the genetic variation of the coding sequences but further work can integrate regulatory elements in 5′ and 3′ regulatory sequences of those encoding genes. The identification of protein variants within the diversity panel will need to be further explored as they may represent adaption to cope with low nutrient availability in natural habitats.

### Strategies to improve nitrogen use efficiency could come up from the identification of the genetic determinism implicated in ethylene production

A significant negative correlation was found between total biomass and ethylene production in Arabidopsis seedlings grown at low nitrate supply, while being opposite in a high nitrate medium (Figure 4). This once more underlines the influence of nutrient supply on ethylene responses, as previously reported by Smalle et al. (1997). Particularly, the effects of mineral nutrient deficiencies or excess on ethylene plant responses must be critically examined. The concept of reducing ethylene production to limit the damages to plants upon biotic and abiotic stresses was already considered, for example in the presence of microbial inoculants with ACC deaminase activity (Gamalero and Glick, 2015). However, manipulating ethylene biosynthetic pathway to improve NUE must be carefully considered and the effectiveness of such improvement must be evaluated (Khan et al., 2015). For instance, ethylene generally down-regulate photosynthetic genes (Van Zhong et al., 2003) but ethylene insensitivity can also lower photosynthetic activity (Tholen et al., 2007). Therefore, ethylene has not to be exclusively seen as a biomass negative regulator and senescence inducer *per se*, since it can also have a positive action on NUE. For instance, delay of leaf senescence may prolong photosynthesis, but rapid senescence increases N remobilization from vegetative parts to sink organs (reviewed in Xu et al., 2012). Furthermore, spraying ethephon- a molecule inducing ethylene release- on *Brassica juncea* can ameliorate photosynthesis rate and NUE (Khan et al., 2008; Iqbal et al., 2011).

### Conclusion and perspectives

This work highlighted the natural variation of ethylene production offered by a small panel of Arabidopsis accessions in response to N supply. The development of analytical procedures allowing the simultaneous measurement of much larger number of gaseous samples will be a key step for successful genome wide association strategies (Atwell et al., 2010) with a higher number of accessions. The output of such studies may support the nomination of *loci* and alleles in the cross-talk between ethylene and mineral nutrition.

## Author contributions

HDG, JDP, CH, and JX performed research and drew figures. HDG and CH wrote the paper and carried additional experiments during the revision. All authors edited the paper.

## Funding

This work is supported by an incentive research grant from the Fonds de la Recherche Scientifique (F.R.S.-FNRS). JDP was PhD research fellow of the Fonds pour la Recherche en Industrie et en Agriculture (FRIA) and CH is an F.R.S.-FNRS research associate. DVS thanks Ghent University for financial support (Bijzonder Onderzoeksfonds project 01B02112).

### Conflict of interest statement

The authors declare that the research was conducted in the absence of any commercial or financial relationships that could be construed as a potential conflict of interest.
