# Supplementary material for: A Comparative Study of Ethylene Emanation upon Nitrogen Deficiency in Natural Accessions of Arabidopsis thaliana
Source: Front Plant Sci. 2016 Feb 10;7:70. doi: 10.3389/fpls.2016.00070 (PMC4748056; doi:10.3389/fpls.2016.00070)
Supplement: Supplementary file 8 [file Image1.PDF]

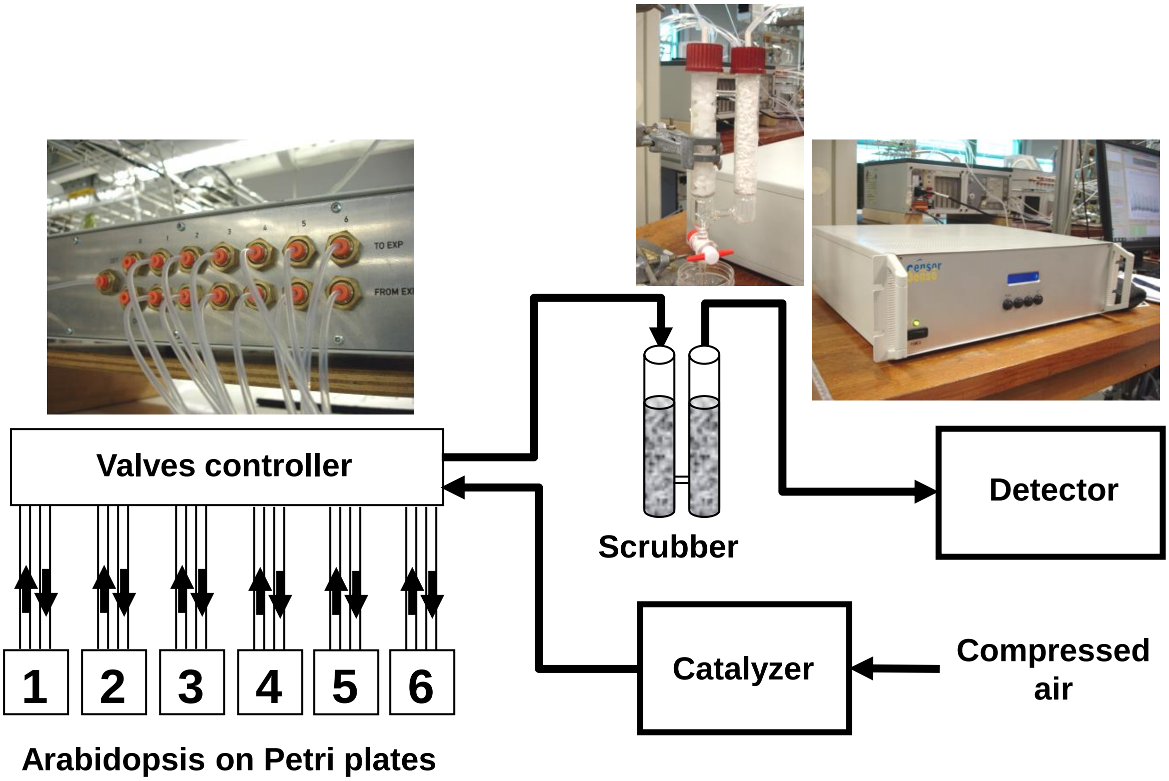

Figure S1 | Experimental setting for measuring ethylene emanation. The circuit is used for measuring 6 samples (Petri plates) in rotation. In that ‘stop and flow’ mode, ethylene is allowed to accumulate for 1 h before it is flushed into the detector. While ethylene from a cuvette is measured during 12 min, no air flow is applied to the other cuvettes, hence optimizing the measurement efficiency. Prior entering the ethylene detector, carbon dioxide and water vapour are eliminated using a scrubber with soda lime and $CaCl_2$.
