# Supplementary material for: A Comparative Study of Ethylene Emanation upon Nitrogen Deficiency in Natural Accessions of Arabidopsis thaliana
Source: Front Plant Sci. 2016 Feb 10;7:70. doi: 10.3389/fpls.2016.00070 (PMC4748056; doi:10.3389/fpls.2016.00070)
Supplement: Supplementary file 9 [file Image2.PDF]

Fig S2 | Amino acid sequence alignment of ACC synthase proteins in Arabidopsis accessions.

The protein sequences of eleven ACS isozymes (note that ASC3 is not presented here because it is a truncated polypeptide) from 21 accessions were retrieved from Salk Arabidopsis 1,001 Genomes database and compared to Col-0 reference accession. Perfect matches are indicated by '.' and deletions by '/'. Synonymous substitutions are indicated in green. Non-synonymous substitutions are highlighted in red, with a black letter indicating a conservative substitution, which maintains the property of the amino acids (acidic polar: D, E; basic polar: H, K, R; neutral polar: N, Q, S, T, Y; neutral slightly polar: C, W; neutral non-polar: A, F, G, I, L, M, P, V) and with a white letter indicating a non-conservative substitution (change between those groups). Seven conserved domains between ACS proteins are marked as boxes 1 to 7. Within box 1, the E residue (except for ACS 10) is involved in substrate specificity. The S residue marked at the end of ACS1-6, 8, 9 and 11 is a putative phosphorylation site. Eleven other residues marked in bold and underlined are conserved among ACS isozymes and various amino transferases. Further description on the motifs can be found in Yamagami et al. (2003).

ACS1

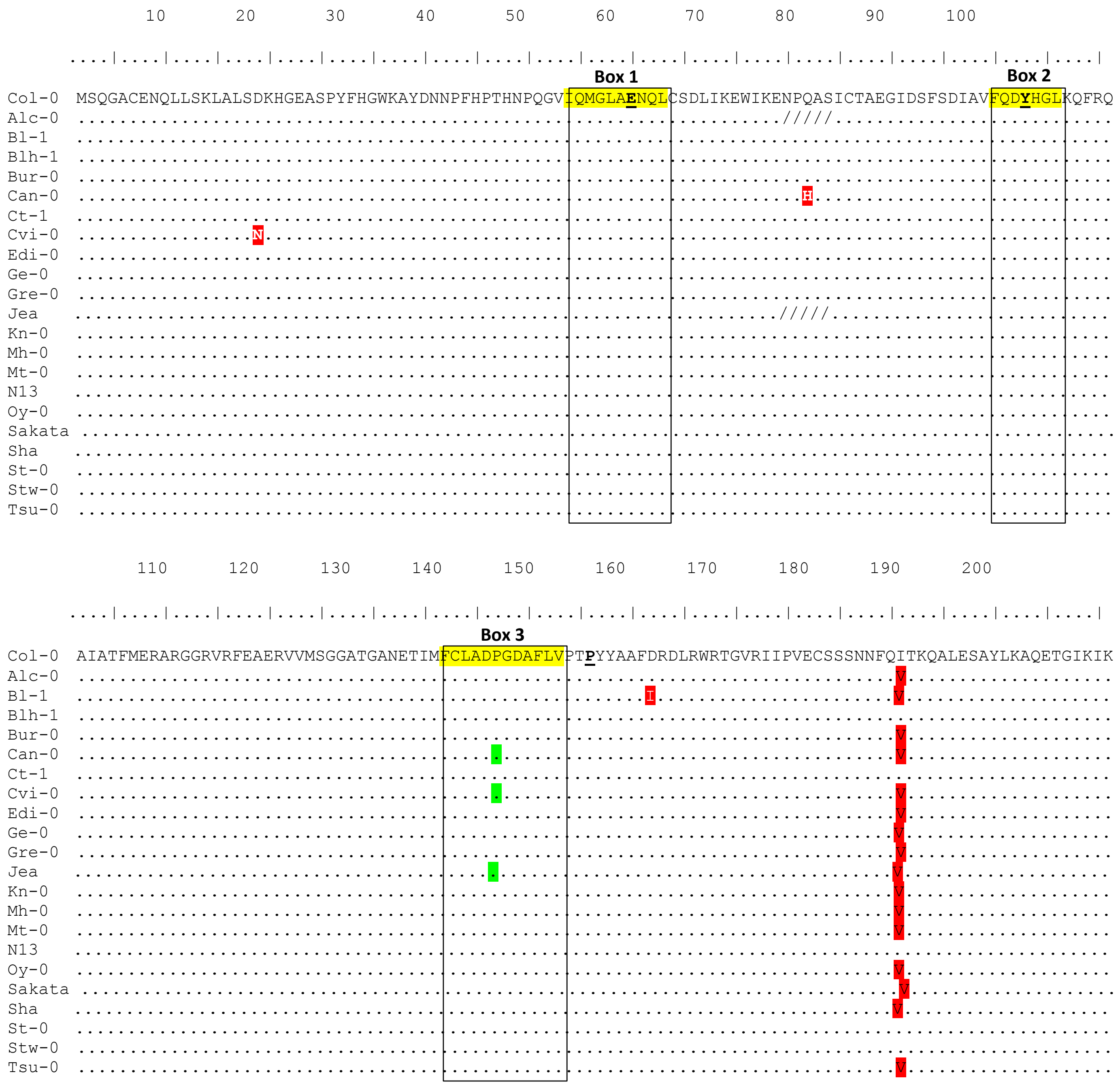
10 20 30 40 50 60 70 80 90 100
Box 1
Box 2
Col-0 MSQGACENQLLSKLALSDKHGEASPYFHGWKAYDNNPFHPTHNPQGVIQMGLAENQLCSDLIKEWIKENPQASICTAEGIDSFSDIAVFQDYHGLKQFRQ
Alc-0
Bl-1
Blh-1
Bur-0
Can-0
Ct-1
Cvi-0
Edi-0
Ge-0
Gre-0
Jea
Kn-0
Mh-0
Mt-0
N13
Oy-0
Sakata
Sha
St-0
Stw-0
Tsu-0
N
H
/////
110 120 130 140 150 160 170 180 190 200
Box 3
Col-0 AIATFMERARGGRVRFEAERVVMSGGATGANETIMFCLADPGDAFLVPTPYYAAFDRDLRWRTGVRIIPVECSSSNNFQITKQALESAYLKAQETGIKIK
I
V

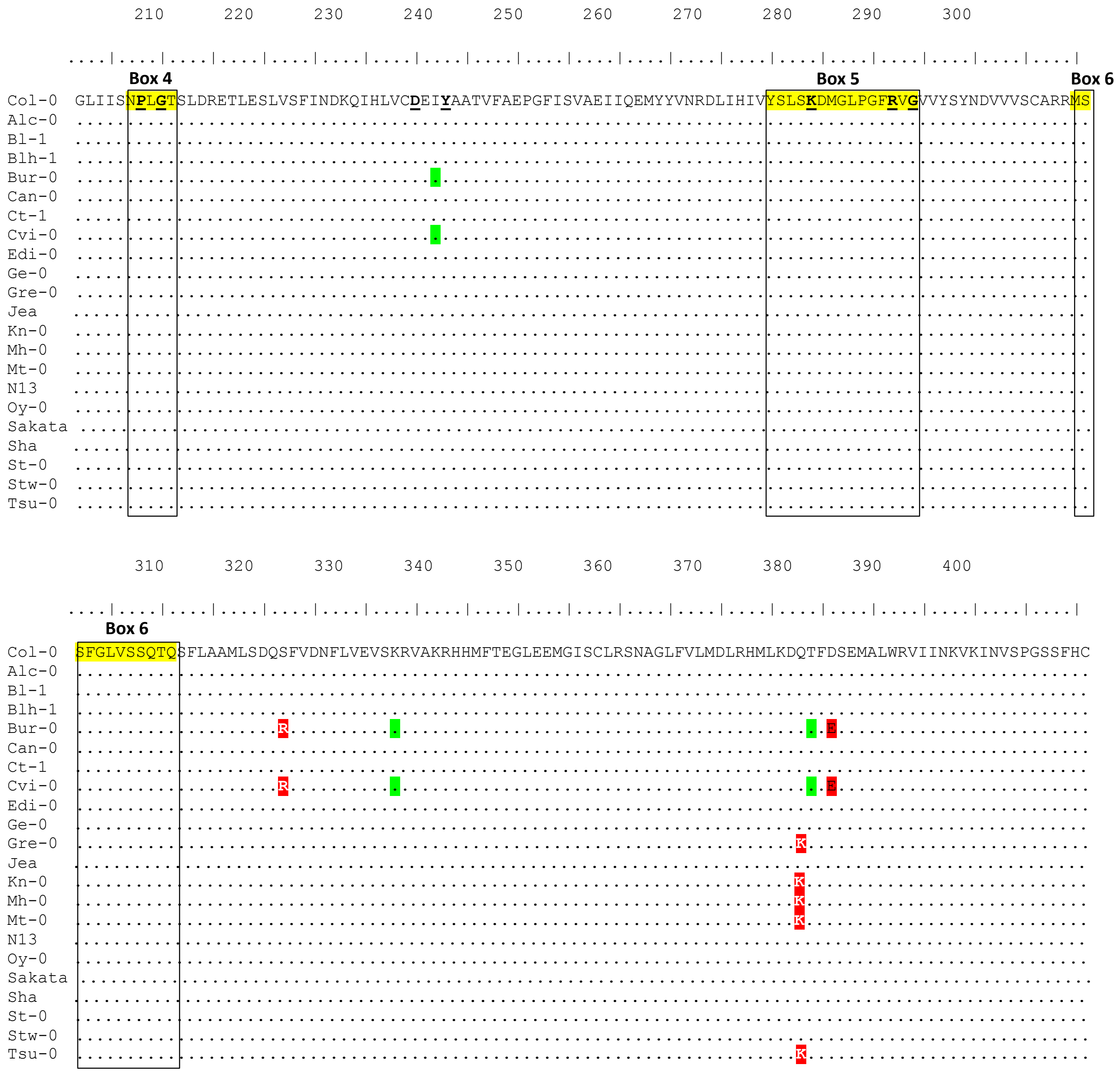
210 220 230 240 250 260 270 280 290 300
Box 4
Box 5
Box 6
Col-0 GLIISNPLGTSLDRETLESLVSFINDKQIHLVCDEIYAATVFAEPGFISVAEIIQEMYYVNRDLIHIVYSLSKDMGLPGFRVGVVYSYNDVVVSCARRMS
Alc-0
Bl-1
Blh-1
Bur-0
Can-0
Ct-1
Cvi-0
Edi-0
Ge-0
Gre-0
Jea
Kn-0
Mh-0
Mt-0
N13
Oy-0
Sakata
Sha
St-0
Stw-0
Tsu-0
310 320 330 340 350 360 370 380 390 400
Box 6
Col-0 SFGLVSSQTQSFLAAMLSDQSFVDNFLVEVSKRVAKRHHMFTEGLEEMGISCLRSNAGLFVLMDLRHMLKDQTFDSEMALWRVIINKVKINVSPGSSFHC
Alc-0
Bl-1
Blh-1
Bur-0 R E
Can-0
Ct-1
Cvi-0 R E
Edi-0
Ge-0
Gre-0 K
Jea
Kn-0 K
Mh-0 K
Mt-0 K
N13
Oy-0
Sakata
Sha
St-0
Stw-0
Tsu-0 K

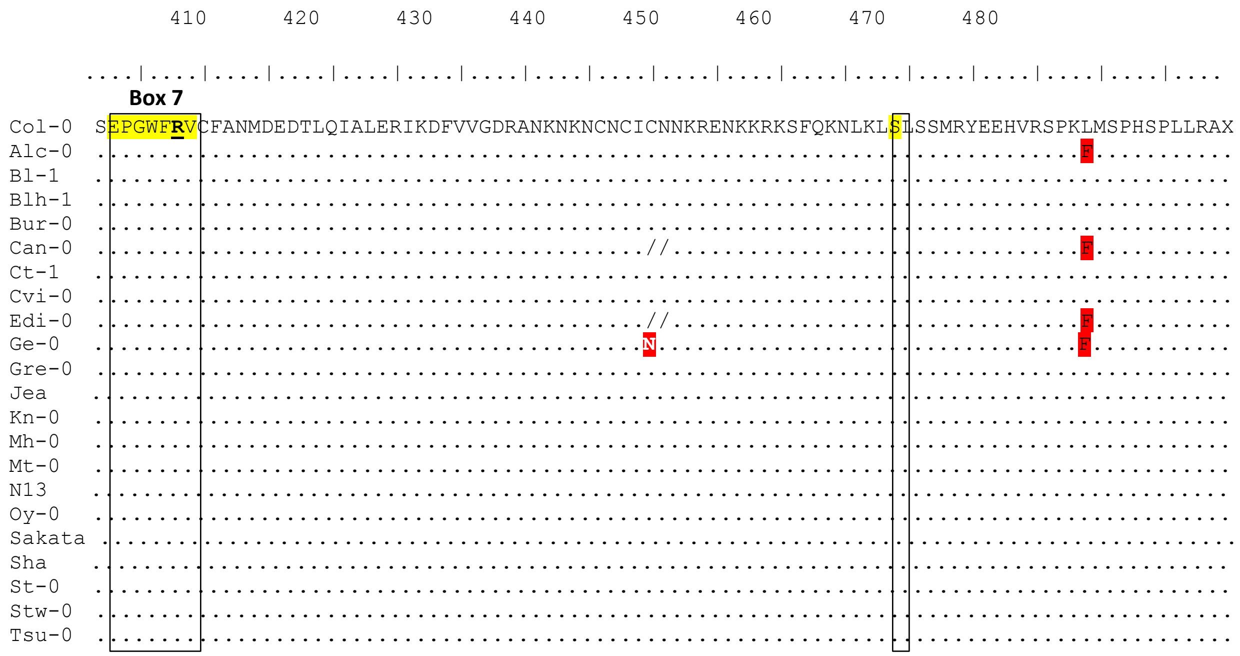

410
420
430
440
450
460
470
480
Box 7
Col-0 SEPGWFRVCFANMDEDTLQIALERIKDFVVGDRANKNKNCNCICNNKRENKKRKSFQKNLKLSLSSMRYEEHVRSPKLMSPHSPLLRAX
Alc-0
F
Bl-1
Blh-1
Bur-0
Can-0
//
F
Ct-1
Cvi-0
Edi-0
//
F
Ge-0
N
F
Gre-0
Jea
Kn-0
Mh-0
Mt-0
N13
Oy-0
Sakata
Sha
St-0
Stw-0
Tsu-0

ACS2

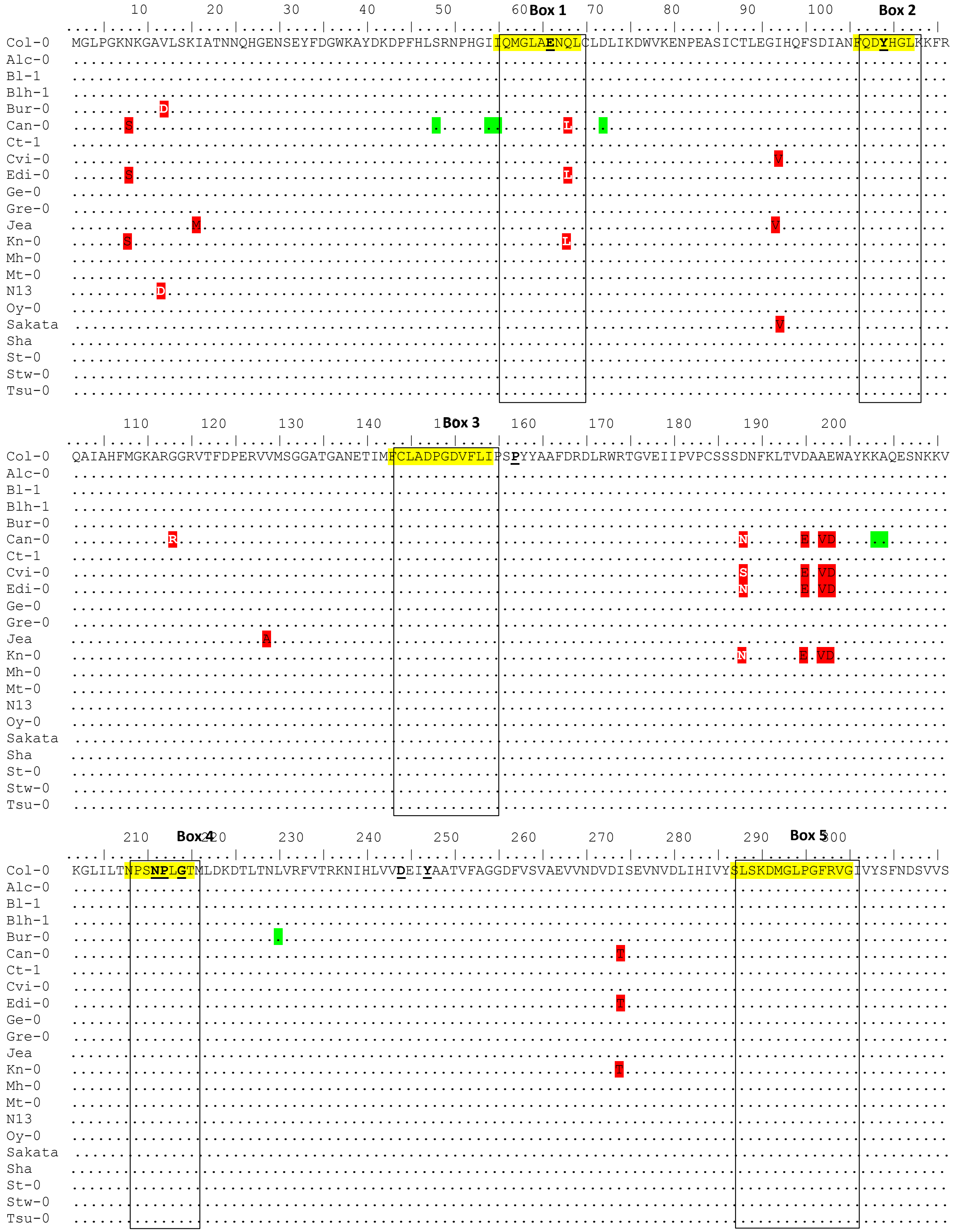
Box 1
Box 2
Box 3
Box 4
Box 5
Col-0 MGLPGKNKGAVLSKIATNNQHGENSEYFDGWKAYDKDPFHLSRNPHGIIQMGLAENQLCLDLIKDWVKENPEASICTLEGIHQFSDIANFQDYHGLKKFR
Col-0 QAIAHFMGKARGGRVTFDPERVVMSGGATGANETIMFCLADPGDVFLIPSPYYAAFDRDLRWRTGVEIIPVPCSSSDNFKLTVDAAEWAYKKAQESNKKV
Col-0 KGLILTNPSNPLGTMLDKDTLTNLVRFVTRKNIHLVVDEIYAATVFAGGDFVSVAEVVNDVDISEVNVDLIHIVYSLSKDMGLPGFRVGIVYSFNDSVVS
Alc-0
Bl-1
Blh-1
Bur-0
Can-0
Ct-1
Cvi-0
Edi-0
Ge-0
Gre-0
Jea
Kn-0
Mh-0
Mt-0
N13
Oy-0
Sakata
Sha
St-0
Stw-0
Tsu-0

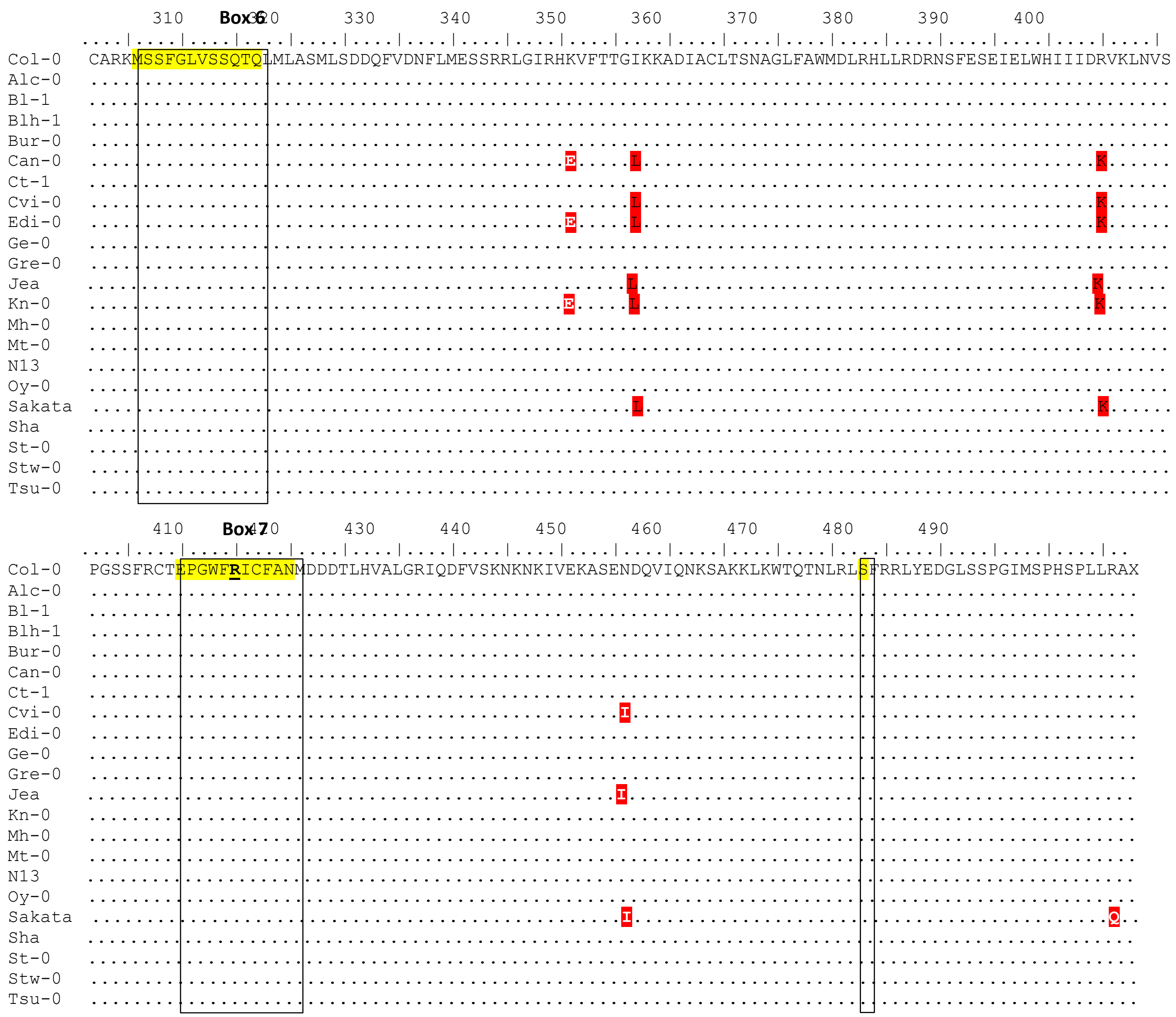

Box 6
310 320 330 340 350 360 370 380 390 400
Col-0 CARKMSSFGLVSSQTQLMLASMLSDDQFVDNFLMESSRRLGIRHKVFTTGIKKADIACLTSNAGLFAWMDLRHLLRDRNSFESEIELWHIIIDRVKLNVS
Alc-0
Bl-1
Blh-1
Bur-0
Can-0 E L K
Ct-1
Cvi-0 L K
Edi-0 E L K
Ge-0
Gre-0
Jea L K
Kn-0 E L K
Mh-0
Mt-0
N13
Oy-0
Sakata L K
Sha
St-0
Stw-0
Tsu-0
Box 7
410 420 430 440 450 460 470 480 490
Col-0 PGSSFRCTEPGWFRICFANMDDDTLHVALGRIQDFVSKNKNKIVEKASENDQVIQNKSAKKLKWTQTNLRLSFRRLYEDGLSSPGIMSPHSPLLRAX
Alc-0
Bl-1
Blh-1
Bur-0
Can-0
Ct-1
Cvi-0 I
Edi-0
Ge-0
Gre-0
Jea I
Kn-0
Mh-0
Mt-0
N13
Oy-0
Sakata I Q
Sha
St-0
Stw-0
Tsu-0

ACS4

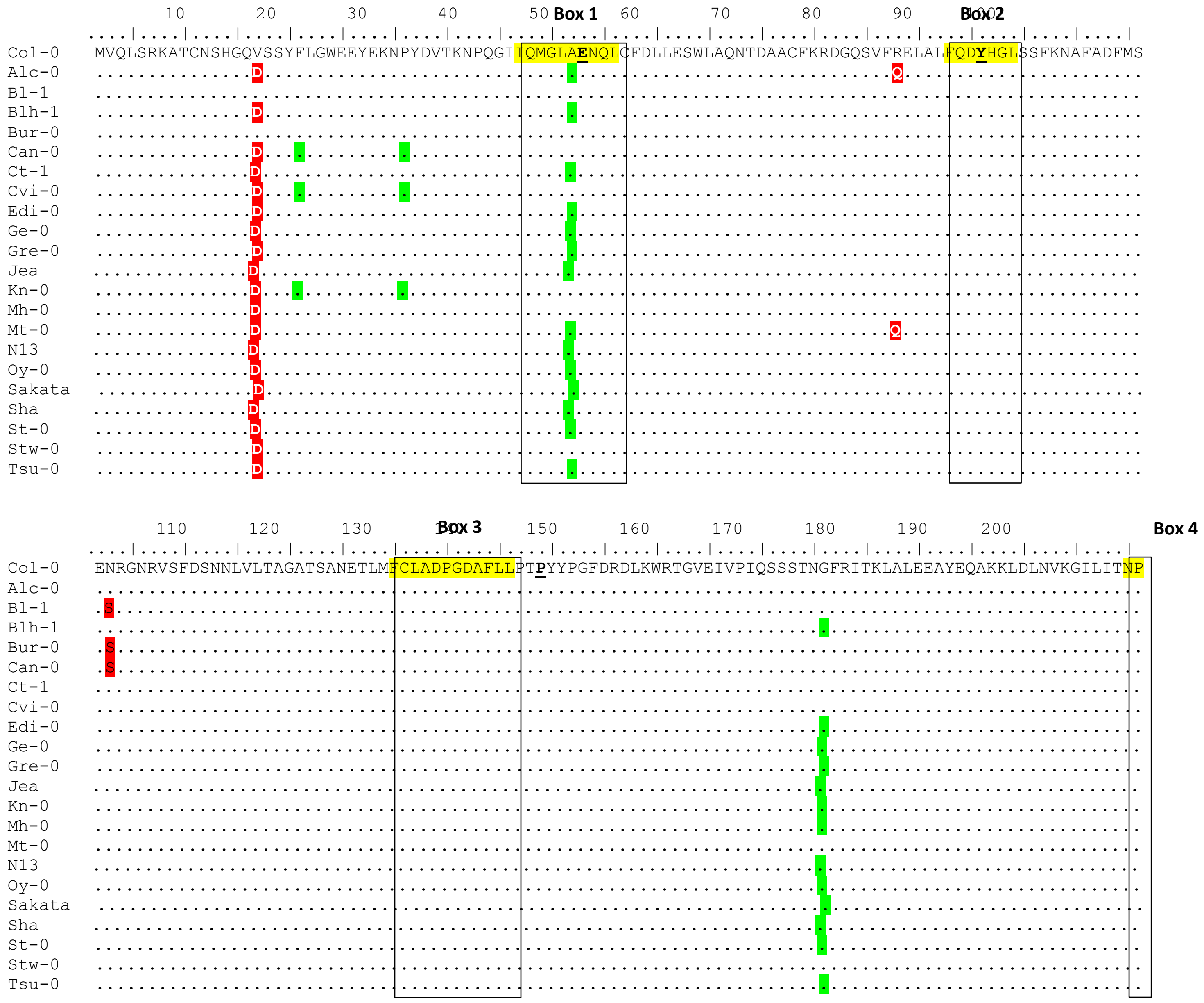
Box 1
Box 2
Box 3
Box 4
Col-0 MVQLSRKATCNSHGQVSSYFLGWEEYEKNPYDVTKNPQGIIQMGLAENQLCFDLLESWLAQNTDAACFKRDGQSVFRELALFQDYHGLSSFKNAFADFMS
Col-0 ENRGNRVSFDSNNLVLTAGATSANETLMFCLADPGDAFLLPTPYYPGFDRDLKWRTGVEIVPIQSSSTNGFRITKLALEEAYEQAKKLDLNVKGILITNP
Alc-0
Bl-1
Blh-1
Bur-0
Can-0
Ct-1
Cvi-0
Edi-0
Ge-0
Gre-0
Jea
Kn-0
Mh-0
Mt-0
N13
Oy-0
Sakata
Sha
St-0
Stw-0
Tsu-0

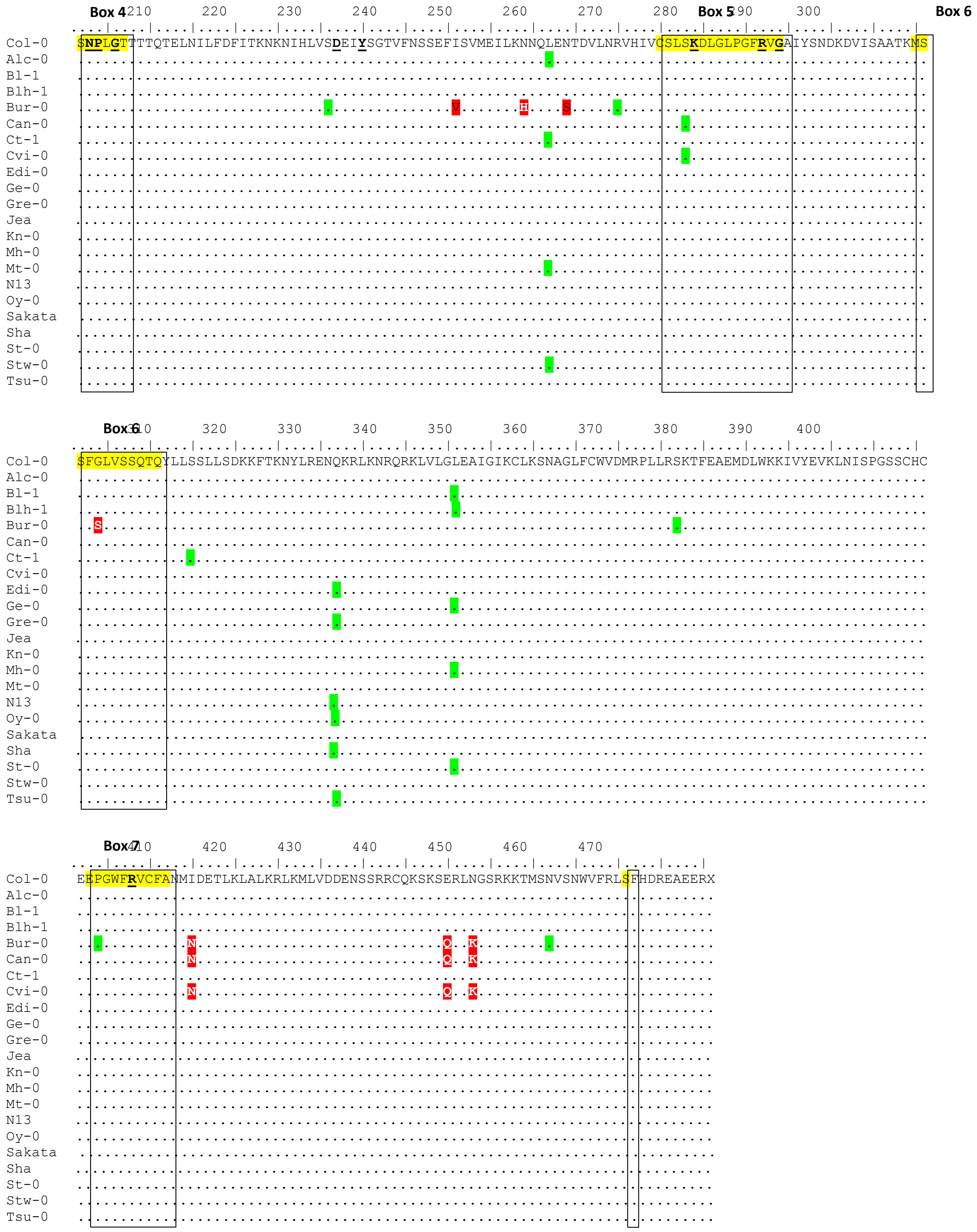

Box 4
Box 5
Box 6
210 220 230 240 250 260 270 280 290 300
Col-0 SNPLGTTTTQTELNILFDFITKNKNIHLVSDEIYSGTVFNSSEFISVMEILKNNQLENTDVLNRVHIVCSLSKDLGLPGFRVGAIYSNDKDVISAATKMS
Alc-0
Bl-1
Blh-1
Bur-0 V H S
Can-0
Ct-1
Cvi-0
Edi-0
Ge-0
Gre-0
Jea
Kn-0
Mh-0
Mt-0
N13
Oy-0
Sakata
Sha
St-0
Stw-0
Tsu-0
Box 6
310 320 330 340 350 360 370 380 390 400
Col-0 SFGLVSSQTQYLLSSLLSDKKFTKNYLRENQKRLKNRQRKLVLGLEAIGIKCLKSNAGLFCWVDMRPLLRSKTFEAEMDLWKKIVYEVKLNISPGSSCHC
Bur-0 S
Box 7
410 420 430 440 450 460 470
Col-0 EEPGWFRVCFANMIDETLKLALKRLKMLVDDENSSRRCQKSKSERLNGSRKKTMSNVSNWVFRLSFHDREAEERX
Bur-0 N Q K
Can-0 N Q K
Cvi-0 N Q K

ACS5

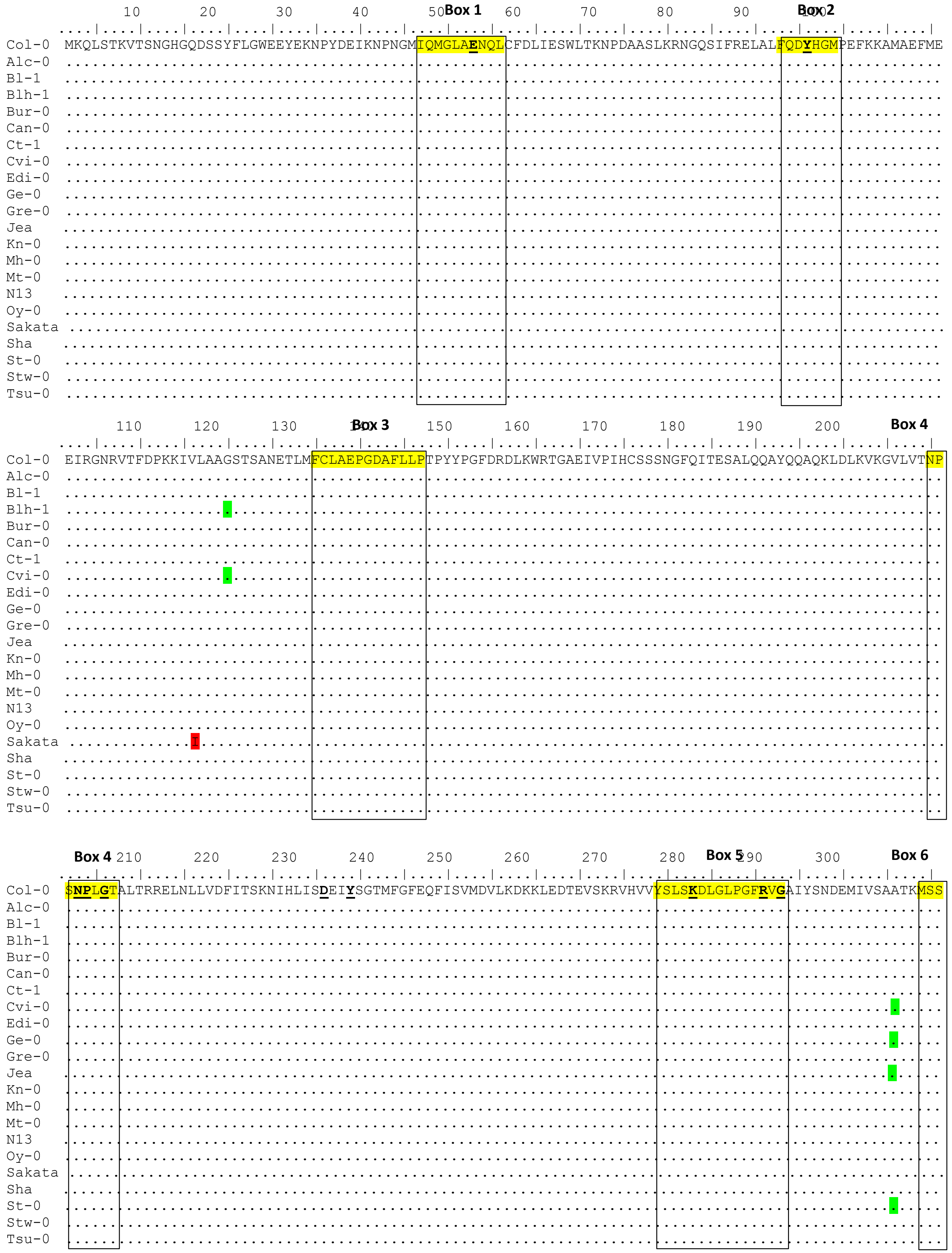

Box 1
Box 2
Col-0 MKQLSTKVTSNGHGQDSSYFLGWEEYEKNPYDEIKNPNGMIQMGLAENQLCFDLIESWLTKNPDAASLKRNGQSIFRELALFQDYHGMPEFKKAMAEFME
Box 3
Box 4
Col-0 EIRGNRVTFDPKKIVLAAGSTSANETLMFCLAEPGDAFLLPTPYYPGFDRDLKWRTGAEIVPIHCSSSNGFQITESALQQAYQQAQKLDLKVKGVLVTNP
Box 4
Box 5
Box 6
Col-0 SNPLGTALTRRELNLLVDFITSKNIHLISDEIYSGTMFGFEQFISVMDVLKDKKLEDTEVSKRVHVVYSLSKDLGLPGFRVGAIYSNDEMIVSAATKMSS
Alc-0
Bl-1
Blh-1
Bur-0
Can-0
Ct-1
Cvi-0
Edi-0
Ge-0
Gre-0
Jea
Kn-0
Mh-0
Mt-0
N13
Oy-0
Sakata
Sha
St-0
Stw-0
Tsu-0
I

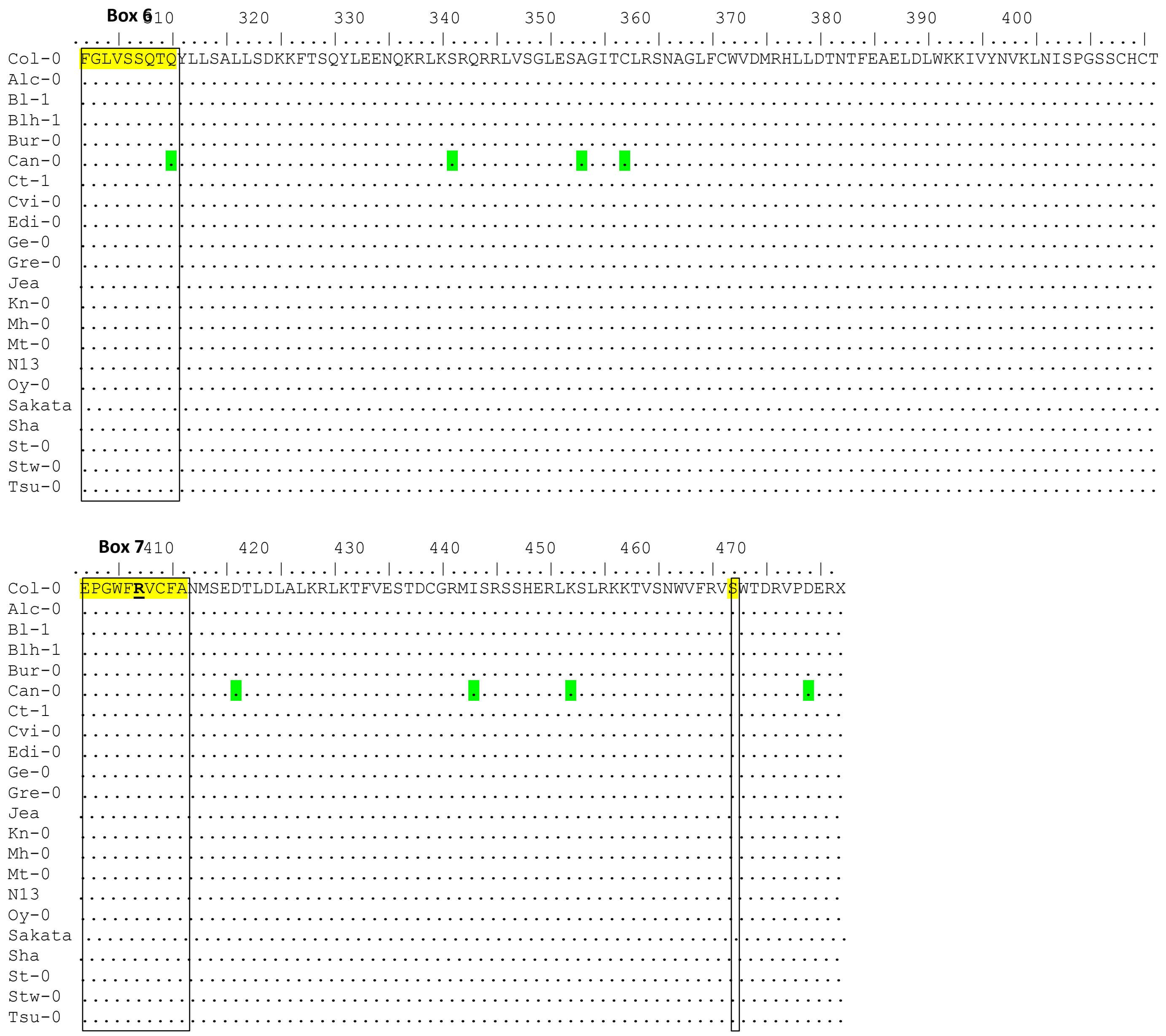
Box 6
310 320 330 340 350 360 370 380 390 400
Col-0 FGLVSSQTQYLLSALLSDKKFTSQYLEENQKRLKSRQRRLVSGLESAGITCLRSNAGLFCWVDMRHLLDTNTFEAELDLWKKIVYNVKLNISPGSSCHCT
Alc-0
Bl-1
Blh-1
Bur-0
Can-0
Ct-1
Cvi-0
Edi-0
Ge-0
Gre-0
Jea
Kn-0
Mh-0
Mt-0
N13
Oy-0
Sakata
Sha
St-0
Stw-0
Tsu-0
Box 7
410 420 430 440 450 460 470
Col-0 EPGWFRVCFANMSEDTLDLALKRLKTFVESTDCGRMISRSSHERLKSLRKKTVSNWVFRVSWTDRVPDERX
Alc-0
Bl-1
Blh-1
Bur-0
Can-0
Ct-1
Cvi-0
Edi-0
Ge-0
Gre-0
Jea
Kn-0
Mh-0
Mt-0
N13
Oy-0
Sakata
Sha
St-0
Stw-0
Tsu-0

ACS6

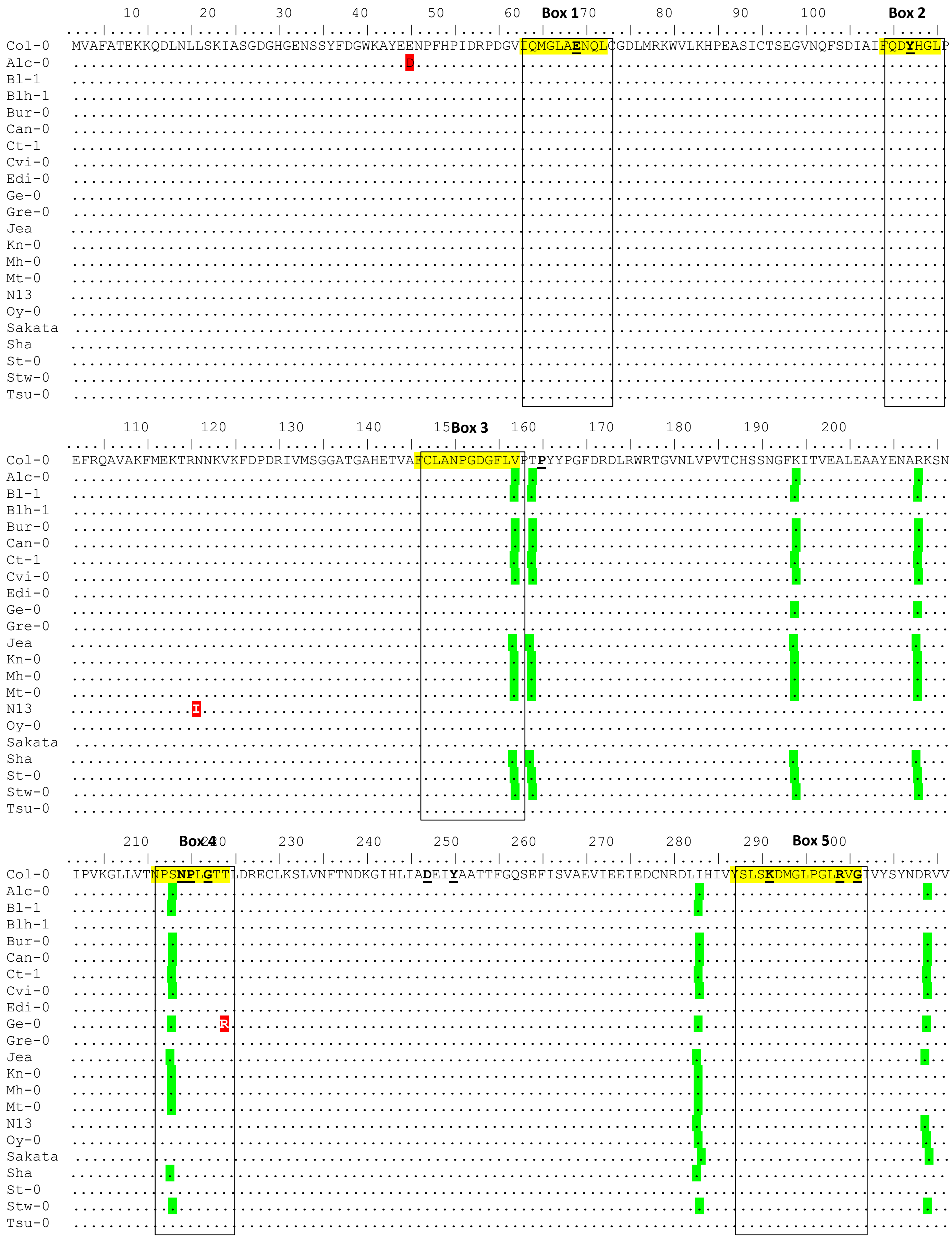
Box 1
Box 2
Box 3
Box 4
Box 5
Col-0 MVAFATEKKQDLNLLSKIASGDGHGENSSYFDGWKAYEENPFHPIDRPDGVIQMGLAENQLCGDLMRKWVLKHPEASICTSEGVNQFSDIAIFQDYHGLP
Col-0 EFRQAVAKFMEKTRNNKVKFDPDRIVMSGGATGAHETVAFCLANPGDGFLVPTPYYPGFDRDLRWRTGVNLVPVTCHSSNGFKITVEALEAAYENARKSN
Col-0 IPVKGLLVTNPSNPLGTTLDRECLKSLVNFTNDKGIHLIADEIYAATTFGQSEFISVAEVIEEIEDCNRDLIHIVYSLSKDMGLPGLRVGIVYSYNDRVV
Alc-0
Bl-1
Blh-1
Bur-0
Can-0
Ct-1
Cvi-0
Edi-0
Ge-0
Gre-0
Jea
Kn-0
Mh-0
Mt-0
N13
Oy-0
Sakata
Sha
St-0
Stw-0
Tsu-0
D
I
R

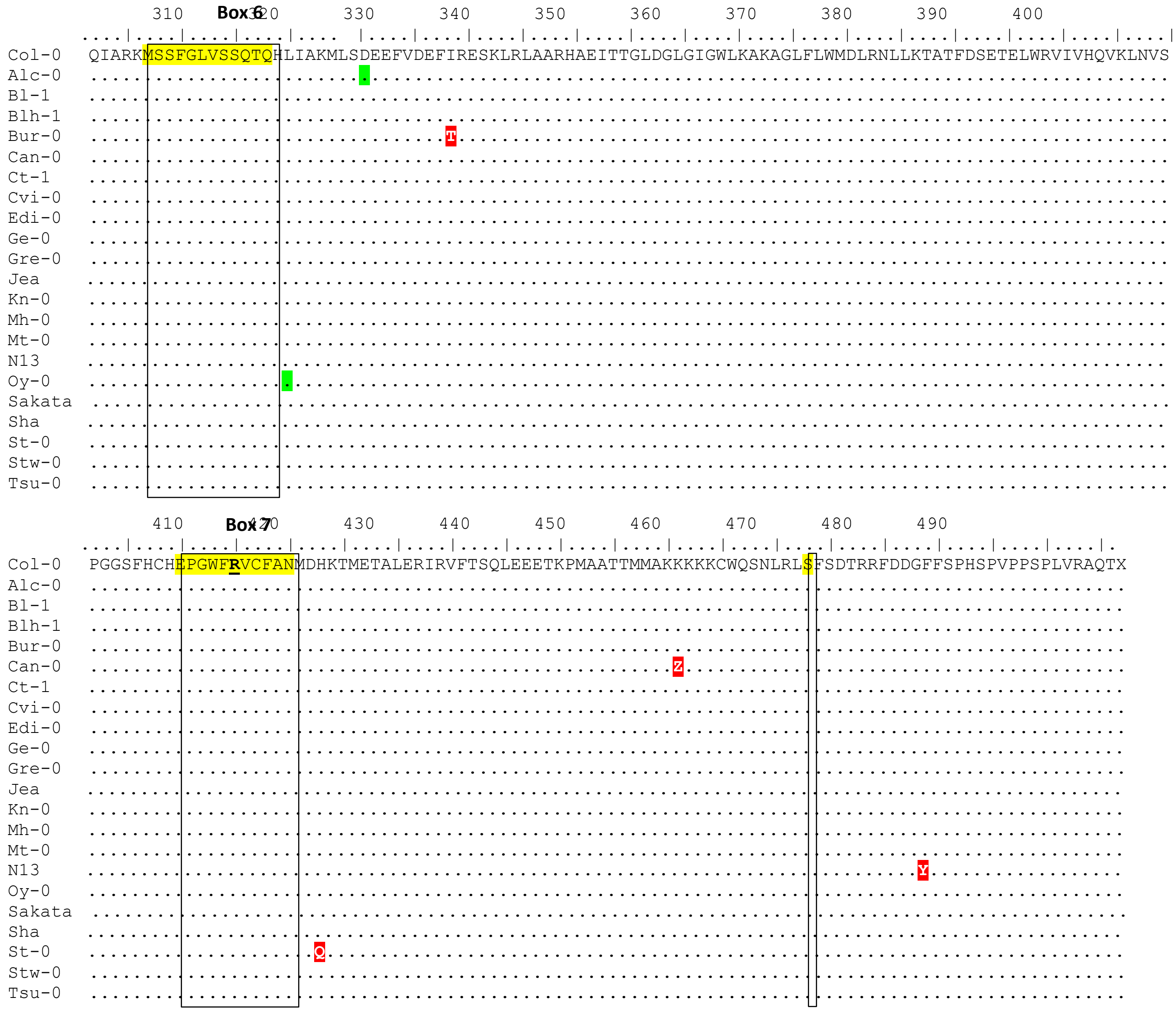

Box 6
310 320 330 340 350 360 370 380 390 400
Col-0 QIARKMSSFGLVSSQTQHLIAKMLSDEEFVDEFIRESKLRLAARHAEITTGLDGLGIGWLKAKAGLFLWMDLRNLLKTATFDSETELWRVIVHQVKLNVS
Alc-0
Bl-1
Blh-1
Bur-0
T
Can-0
Ct-1
Cvi-0
Edi-0
Ge-0
Gre-0
Jea
Kn-0
Mh-0
Mt-0
N13
Oy-0
Sakata
Sha
St-0
Stw-0
Tsu-0
Box 7
410 420 430 440 450 460 470 480 490
Col-0 PGGSFHCHEPGWFRVCFANMDHKTMETALERIRVFTSQLEEETKPMAATTMMAKKKKKCWQSNLRLSFSDTRRFDDGFFSPHSPVPPSPLVRAQTX
Alc-0
Bl-1
Blh-1
Bur-0
Can-0
Z
Ct-1
Cvi-0
Edi-0
Ge-0
Gre-0
Jea
Kn-0
Mh-0
Mt-0
N13
Y
Oy-0
Sakata
Sha
St-0
Q
Stw-0
Tsu-0

ACS7

```
                10        20        30        40        50        60        70        80        90        100
                                                                        Box 1                              Box 2
Col-0   MGLPLMMERSSNNNNVELSRVAVSDTHGEDSPYFAGWKAYDENPYDESHNPSGVIQMGLAENQVSFDLLETYLEKKNPEGSMWGSKGAPGFRENALFQDY
Alc-0   ....................................................................................................
Bl-1    ....................................................................................................
Blh-1   ....................................................................................................
Bur-0   ....................................................................................................
Can-0   ....................................................................................................
Ct-1    ....................................................................................................
Cvi-0   ....................................................................................................
Edi-0   ....................................................................................................
Ge-0    ....................................................................................................
Gre-0   ....................................................................................................
Jea     ....................................................................................................
Kn-0    ....................................................................................................
Mh-0    ....................................................................................................
Mt-0    ....................................................................................................
N13     ....................................................................................................
Oy-0    ....................................................................................................
Sakata  ....................................................................................................
Sha     ....................................................................................................
St-0    ....................................................................................................
Stw-0   ....................................................................................................
Tsu-0   ....................................................................................................

                110       120       130       140       150       160       170       180       190       200
      Box 2                                        Box 3
Col-0   HGLKTFRQAMASFMEQIRGGKARFDPDRIVLTAGATAANELLTFILADPNDALLVPTPYYPGFDRDLRWRTGVKIVPIHCDSSNHFQITPEALESAYQTA
Alc-0   ....................................................................................................
Bl-1    ....................................................................................................
Blh-1   ....................................................................................................
Bur-0   ....................................................................................................
Can-0   ....................................................................................................
Ct-1    .............................................................................................D......
Cvi-0   ....................................................................................................
Edi-0   ....................................................................................................
Ge-0    ....................................................................................................
Gre-0   ....................................................................................................
Jea     ....................................................................................................
Kn-0    ....................................................................................................
Mh-0    ....................................................................................................
Mt-0    .............................................................................................D......
N13     ....................................................................................................
Oy-0    ....................................................................................................
Sakata  ....................................................................................................
Sha     ....................................................................................................
St-0    ....................................................................................................
Stw-0   ....................................................................................................
Tsu-0   .............................................................................................D......
```

```
              210       220       230       240       250       260       270       280       290       300
      ....|....|....|....|....|.  ...|....|....|....|....|....|....|....|....|....|....|....|....|....|....|
                   Box 4                                                                            Box 5
Col-0  RDANIRVRGVLITNPSNPLGATVQKKVLEDLLDFCVRKNIHLVSDEIYSGSVFHASEFTSVAEIVENIDDVSVKERVHIVYSLSKDLGLPGFRVGTIYSY
Alc-0  ....................................................................................................
Bl-1   ....................................................................................................
Blh-1  ....................................................................................................
Bur-0  ....................................................................................................
Can-0  ....................................................................................................
Ct-1   ....................................................................................................
Cvi-0  ....................................................................................................
Edi-0  ....................................................................................................
Ge-0   ....................................................................................................
Gre-0  ....................................................................................................
Jea    ....................................................................................................
Kn-0   ....................................................................................................
Mh-0   ....................................................................................................
Mt-0   ....................................................................................................
N13    ....................................................................................................
Oy-0   ....................................................................................................
Sakata ....................................................................................................
Sha    ....................................................................................................
St-0   ....................................................................................................
Stw-0  ....................................................................................................
Tsu-0  ....................................................................................................

              310       320       330       340       350       360       370       380       390       400
      ....|....|....|....|....|..  ..|....|....|....|....|....|....|....|....|....|....|....|....|....|....|
                Box 6
Col-0  NDNVVRTARRMSSFTLVSSQTQHMLASMLSDEEFTEKYIRINRERLRRRYDTIVEGLKKAGIECLKGNAGLFCWMNLGFLLEKKTKDGELQLWDVILKEL
Alc-0  ....................................................................................................
Bl-1   ....................................................................................................
Blh-1  ....................................................................................................
Bur-0  ....................................................................................................
Can-0  ....................................................................................................
Ct-1   ....................................................................................................
Cvi-0  ....................................................................................................
Edi-0  ....................................................................................................
Ge-0   ....................................................................................................
Gre-0  ....................................................................................................
Jea    ....................................................................................................
Kn-0   ....................................................................................................
Mh-0   ....................................................................................................
Mt-0   ....................................................................................................
N13    ....................................................................................................
Oy-0   ....................................................................................................
Sakata ....................................................................................................
Sha    ....................................................................................................
St-0   ....................................................................................................
Stw-0  ....................................................................................................
Tsu-0  ....................................................................................................
```

```
                   410        420        430        440
              ....|....|....|....|....|....|....|....|....|..
                                 Box 7
Col-0     NLNISPGSSCHCSEVGWFRVCFANMSENTLEIALKRIHEFMDRRRRFX
Alc-0     K...............................................
Bl-1      ................................................
Blh-1     K...............................................
Bur-0     ................................................
Can-0     K...............................................
Ct-1      K...............................................
Cvi-0     ................................................
Edi-0     K...............................................
Ge-0      K...............................................
Gre-0     K...............................................
Jea       K...............................................
Kn-0      K...............................................
Mh-0      ................................................
Mt-0      K...............................................
N13       K...............................................
Oy-0      K...............................................
Sakata    K...............................................
Sha       K...............................................
St-0      K...............................................
Stw-0     K...............................................
Tsu-0     K...............................................
```

ACS8

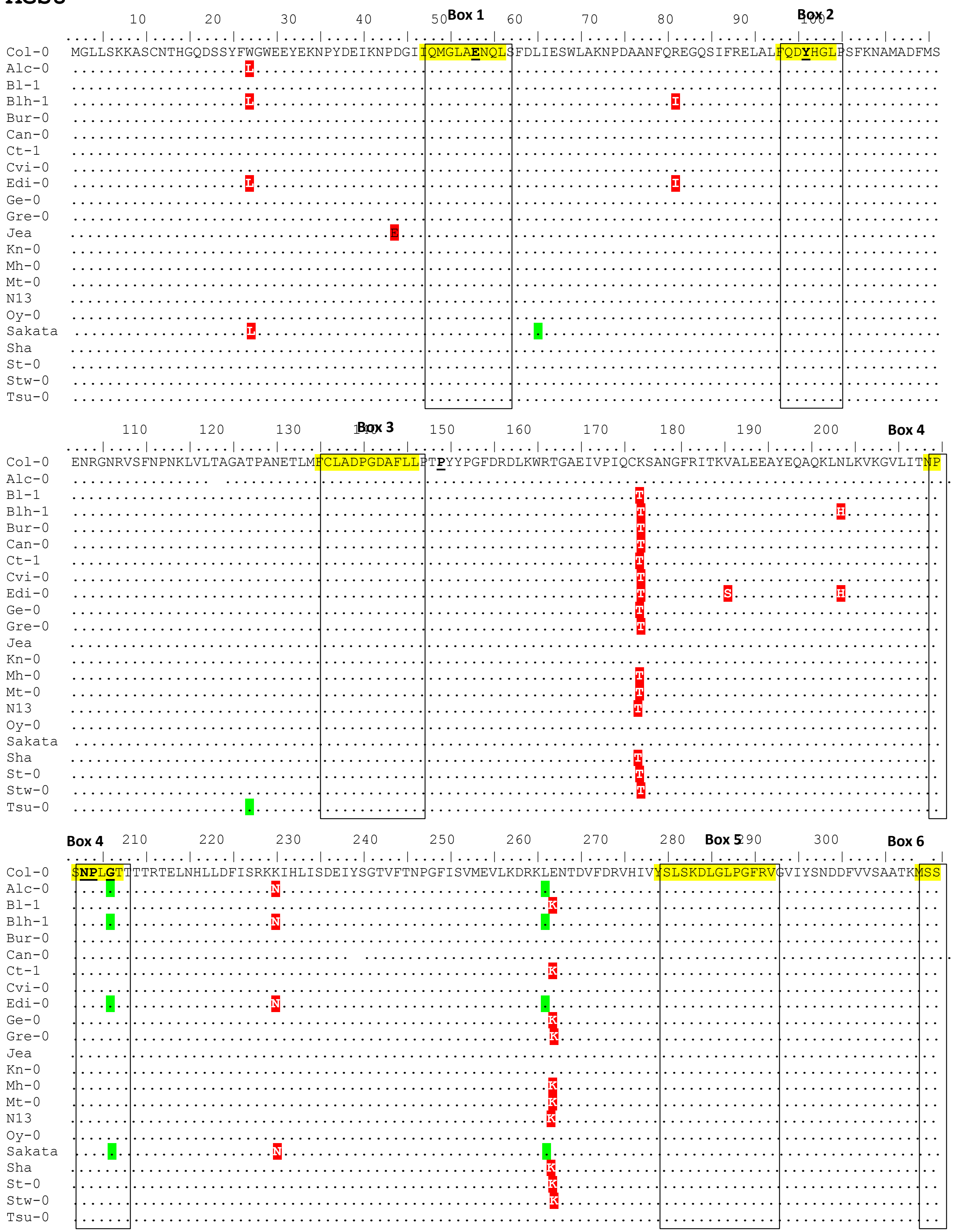
Box 1
Box 2
Box 3
Box 4
Box 5
Box 6
Col-0 MGLLSKKASCNTHGQDSSYFWGWEEYEKNPYDEIKNPDGIIQMGLAENQLSFDLIESWLAKNPDAANFQREGQSIFRELALFQDYHGLPSFKNAMADFMS
Col-0 ENRGNRVSFNPNKLVLTAGATPANETLMFCLADPGDAFLLPTPYYPGFDRDLKWRTGAEIVPIQCKSANGFRITKVALEEAYEQAQKLNLKVKGVLITNP
Col-0 SNPLGTTTTRTELNHLLDFISRKKIHLISDEIYSGTVFTNPGFISVMEVLKDRKLENTDVFDRVHIVYSLSKDLGLPGFRVGVIYSNDDFVVSAATKMSS
Alc-0
Bl-1
Blh-1
Bur-0
Can-0
Ct-1
Cvi-0
Edi-0
Ge-0
Gre-0
Jea
Kn-0
Mh-0
Mt-0
N13
Oy-0
Sakata
Sha
St-0
Stw-0
Tsu-0

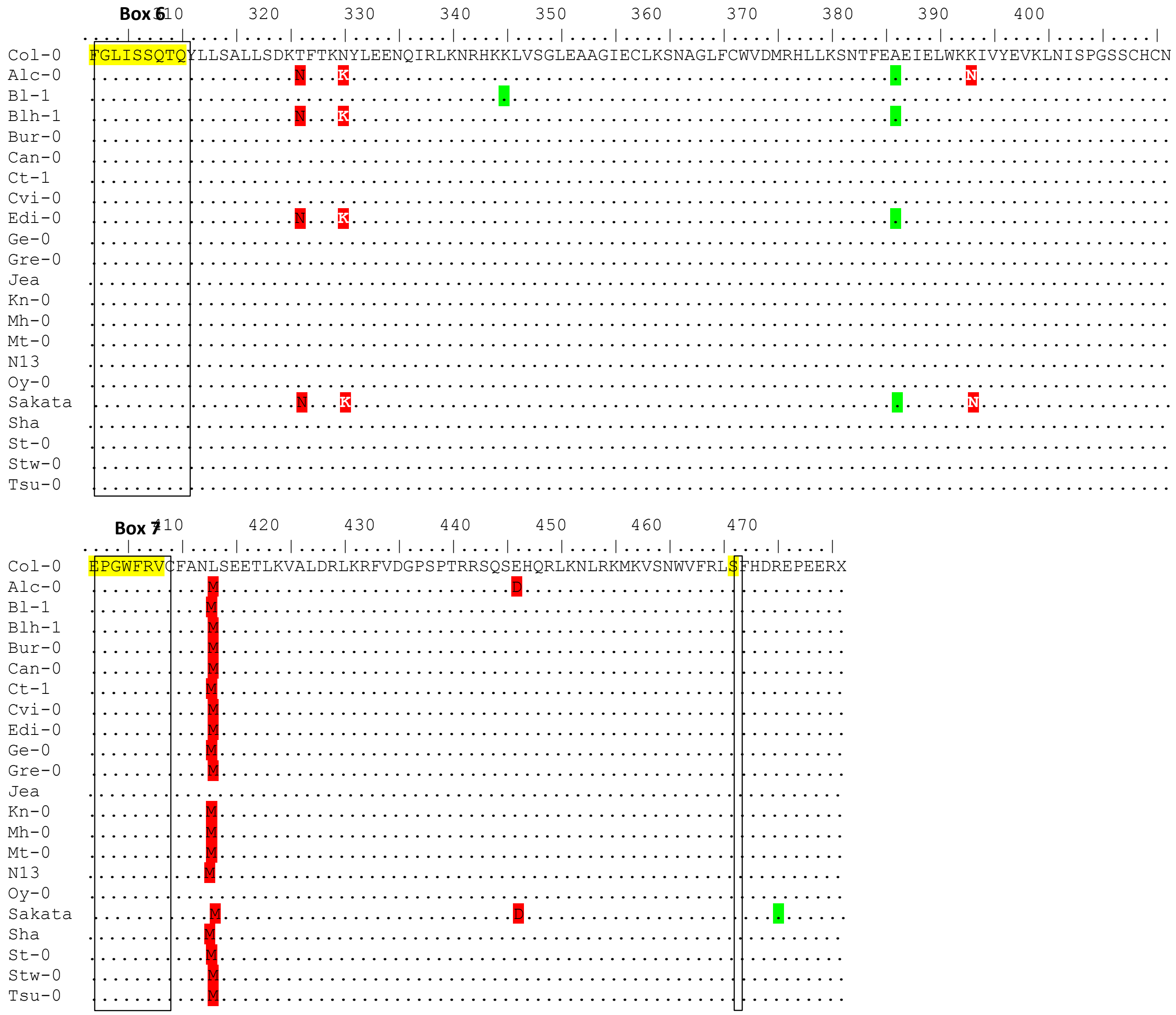

Box 6
310 320 330 340 350 360 370 380 390 400
Col-0 FGLISSQTQYLLSALLSDKTFTKNYLEENQIRLKNRHKKLVSGLEAAGIECLKSNAGLFCWVDMRHLLKSNTFEAEIELWKKIVYEVKLNISPGSSCHCN
Alc-0
Bl-1
Blh-1
Bur-0
Can-0
Ct-1
Cvi-0
Edi-0
Ge-0
Gre-0
Jea
Kn-0
Mh-0
Mt-0
N13
Oy-0
Sakata
Sha
St-0
Stw-0
Tsu-0
Box 7
410 420 430 440 450 460 470
Col-0 EPGWFRVCFANLSEETLKVALDRLKRFVDGPSPTRRSQSEHQRLKNLRKMKVSNWVFRLSFHDREPEERX
Alc-0
Bl-1
Blh-1
Bur-0
Can-0
Ct-1
Cvi-0
Edi-0
Ge-0
Gre-0
Jea
Kn-0
Mh-0
Mt-0
N13
Oy-0
Sakata
Sha
St-0
Stw-0
Tsu-0

ACS9

```
                10        20        30        40        50        60        70        80        90       100
       ....|....|....|....|....|....|....|....|....|....|....|....|....|....|....|....|....|....|....|....|
                                                      Box 1                                  Box 2
Col-0  MKQLSRKVTSNAHGQDSSYFLGWEEYEKNPYDEIKNPNGIIQMGLAENQLCFDLIETWLAKNPDAAGLKKDGQSIFKELALFQDYHGLPEFKKALAEFME
Alc-0  ....................................................................................................
Bl-1   ....................................................................................................
Blh-1  ....................................................................................................
Bur-0  ....................................................................................................
Can-0  ....................................................................................................
Ct-1   ....................................................................................................
Cvi-0  ....................................................................................................
Edi-0  ....................................................................................................
Ge-0   ....................................................................................................
Gre-0  .............................L......................................................................
Jea    ....................................................................................................
Kn-0   ....................................................................................................
Mh-0   .............................L......................................................................
Mt-0   .............................L......................................................................
N13    ....................................................................................................
Oy-0   ........................................................S...........................................
Sakata ....................................................................................................
Sha    ....................................................................................................
St-0   ....................................................................................................
Stw-0  ....................................................................................................
Tsu-0  ....................................................................................................

               110       120       130       140       150       160       170       180       190       200
       ....|....|....|....|....|....|....|....|....|....|....|....|....|....|....|....|....|....|....|....|
                                          Box 3                                                       Box 4
Col-0  EIRGNRVTFDPSKIVLAAGSTSANETLMFCLAEPGDAFLLPTPYYPGFDRDLKWRTGAEIVPIHCSSSNGFQITESALQQAYQQAQKLDLKVKGVLVTNP
Alc-0  ....................................................................................................
Bl-1   ....................................................................................................
Blh-1  ....................................................................................................
Bur-0  ....................................................................................................
Can-0  ....................................................................................................
Ct-1   ....................................................................................................
Cvi-0  ....................................................................................................
Edi-0  ....................................................................................................
Ge-0   ....................................................................................................
Gre-0  ....................................................................................................
Jea    ....................................................................................................
Kn-0   ....................................................................................................
Mh-0   ....................................................................................................
Mt-0   ....................................................................................................
N13    ....................................................................................................
Oy-0   ....................................................................................................
Sakata ...................................................................A................................
Sha    ....................................................................................................
St-0   ....................................................................................................
Stw-0  ....................................................................................................
Tsu-0  ....................................................................................................
```

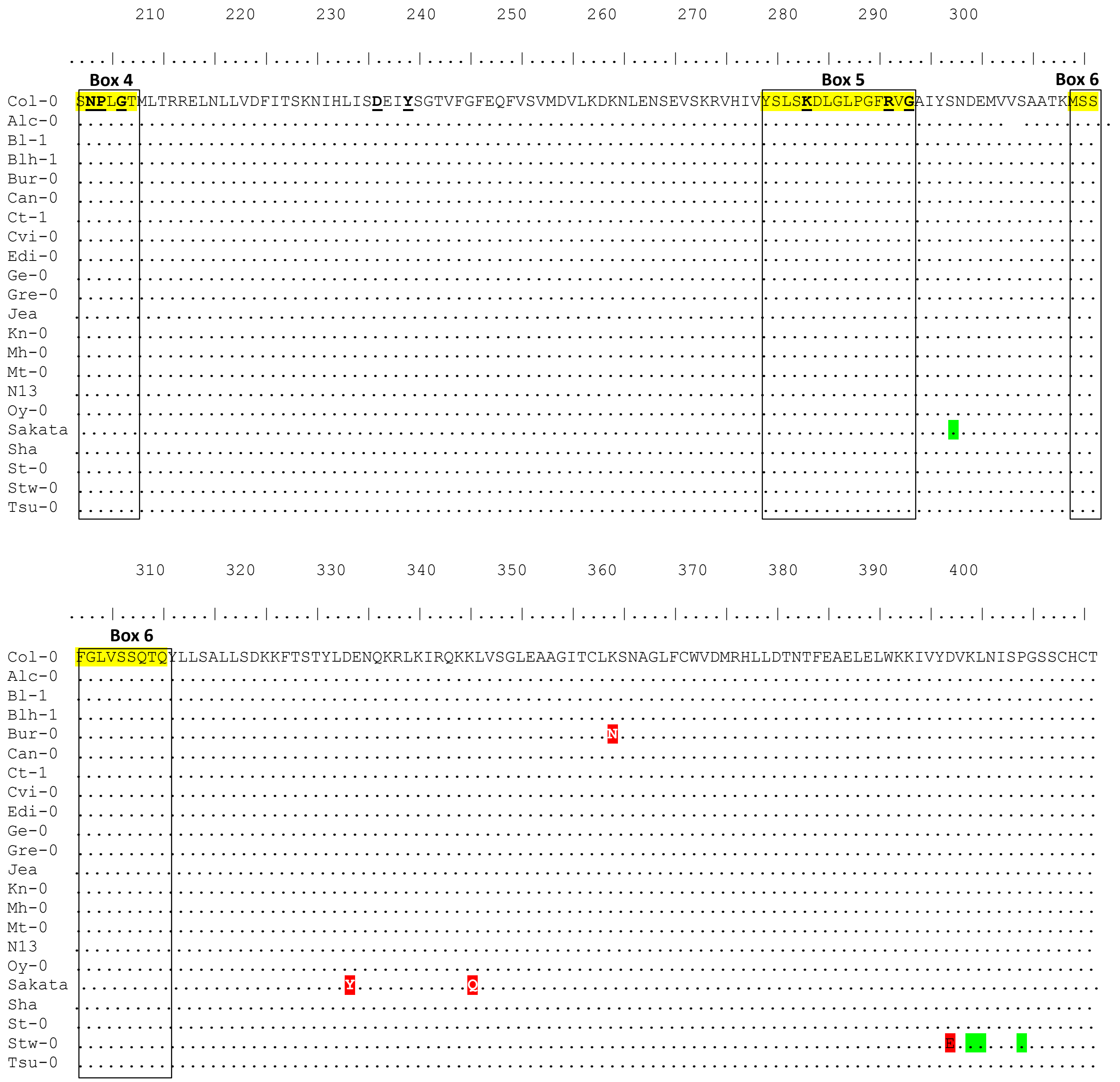
210 220 230 240 250 260 270 280 290 300
Box 4
Box 5
Box 6
Col-0 SNPLGTMLTRRELNLLVDFITSKNIHLISDEIYSGTVFGFEQFVSVMDVLKDKNLENSEVSKRVHIVYSLSKDLGLPGFRVGAIYSNDEMVVSAATKMSS
Alc-0
Bl-1
Blh-1
Bur-0
Can-0
Ct-1
Cvi-0
Edi-0
Ge-0
Gre-0
Jea
Kn-0
Mh-0
Mt-0
N13
Oy-0
Sakata
Sha
St-0
Stw-0
Tsu-0
310 320 330 340 350 360 370 380 390 400
Box 6
Col-0 FGLVSSQTQYLLSALLSDKKFTSTYLDENQKRLKIRQKKLVSGLEAAGITCLKSNAGLFCWVDMRHLLDTNTFEAELELWKKIVYDVKLNISPGSSCHCT
N
Y
Q
E

```
             410        420        430        440        450        460        470
        ....|....|....|....|....|....|....|....|....|....|....|....|....|....|.
         Box 7
Col-0   EPGWFRVCFANMSEDTLDLAMKRLKEYVESTDSRRVISKSSHDRIKSLRKRTVSNWVFRVSWTDRVPDERX
Alc-0   .......................................................................
Bl-1    .......................................................................
Blh-1   .......................................................................
Bur-0   .......................................................................
Can-0   .......................................................................
Ct-1    .......................................................................
Cvi-0   .......................................................................
Edi-0   ...............................I.......................................
Ge-0    .......................................................................
Gre-0   .......................................................................
Jea     .......................................................................
Kn-0    .......................................................................
Mh-0    .......................................................................
Mt-0    .......................................................................
N13     .......................................................................
Oy-0    .......................................................................
Sakata  .......................................................................
Sha     .......................................................................
St-0    .......................................................................
Stw-0   .......................................................................
Tsu-0   .......................................................................
```

ACS10

```
          10        20        30        40        50        60        70        80        90       100
....|....|....|....|....|....|....|....|....|....|....|....|....|....|....|....|....|....|....|....|

Col-0  MTRTEPNRSRSSNSDSDKNSGNVGGGRTTGMRVIVPLQGVVQGRGGLFLGSVIPCAFFYFLQFYLKRNRKNDESDNSGEQNSSASSSSSPNSGLPDPTRS
Alc-0  ....................................................................................................
Bl-1   ....................................................................................................
Blh-1  ....................................................................................................
Bur-0  ....................................................................................................
Can-0  .........W............G.....A.......................................................................
Ct-1   ....................................................................................................
Cvi-0  .....................K..............................................................................
Edi-0  ....................................................................................................
Ge-0   ....................................................................................................
Gre-0  ....................................................................................................
Jea    ....................................................................................................
Kn-0   ....................................................................................................
Mh-0   ....................................................................................................
Mt-0   .........W..........................................................................................
N13    ....................................................................................................
Oy-0   ....................................................................................................
Sakata ......................................................................S.............................
Sha    ....................................................................................................
St-0   ....................................................................................................
Stw-0  ....................................................................................................
Tsu-0  ....................................................................................................

         110       120       130       140       150       160       170       180       190       200
....|....|....|....|....|....|....|....|....|....|....|....|....|....|....|....|....|....|....|....|
                                                                               Box 1
Col-0  QSAGHLTELTGLPRSLSRILLSPRNSGGAVSVSGRVNCVLKGGDSSPYYVGQKRVEDDPYDELGNPDGVIQLGLAQNNKLSLDDWVLENPKEAISDGLSI
Alc-0  ....................................................................................................
Bl-1   ....................................................................................................
Blh-1  ....................................................................................................
Bur-0  ....................................................................................................
Can-0  ....................................................................................................
Ct-1   ....................................................................................................
Cvi-0  ....................................................................................................
Edi-0  ....................................................................................................
Ge-0   ....................................................................................................
Gre-0  ....................................................................................................
Jea    ....................................................................................................
Kn-0   ....................................................................................................
Mh-0   ....................................................................................................
Mt-0   ....................................................................................................
N13    ....................................................................................................
Oy-0   ....................................................................................................
Sakata ....................................................................................................
Sha    ...............................L....................................................................
St-0   ....................................................................................................
Stw-0  ....................................................................................................
Tsu-0  ....................................................................................................
```

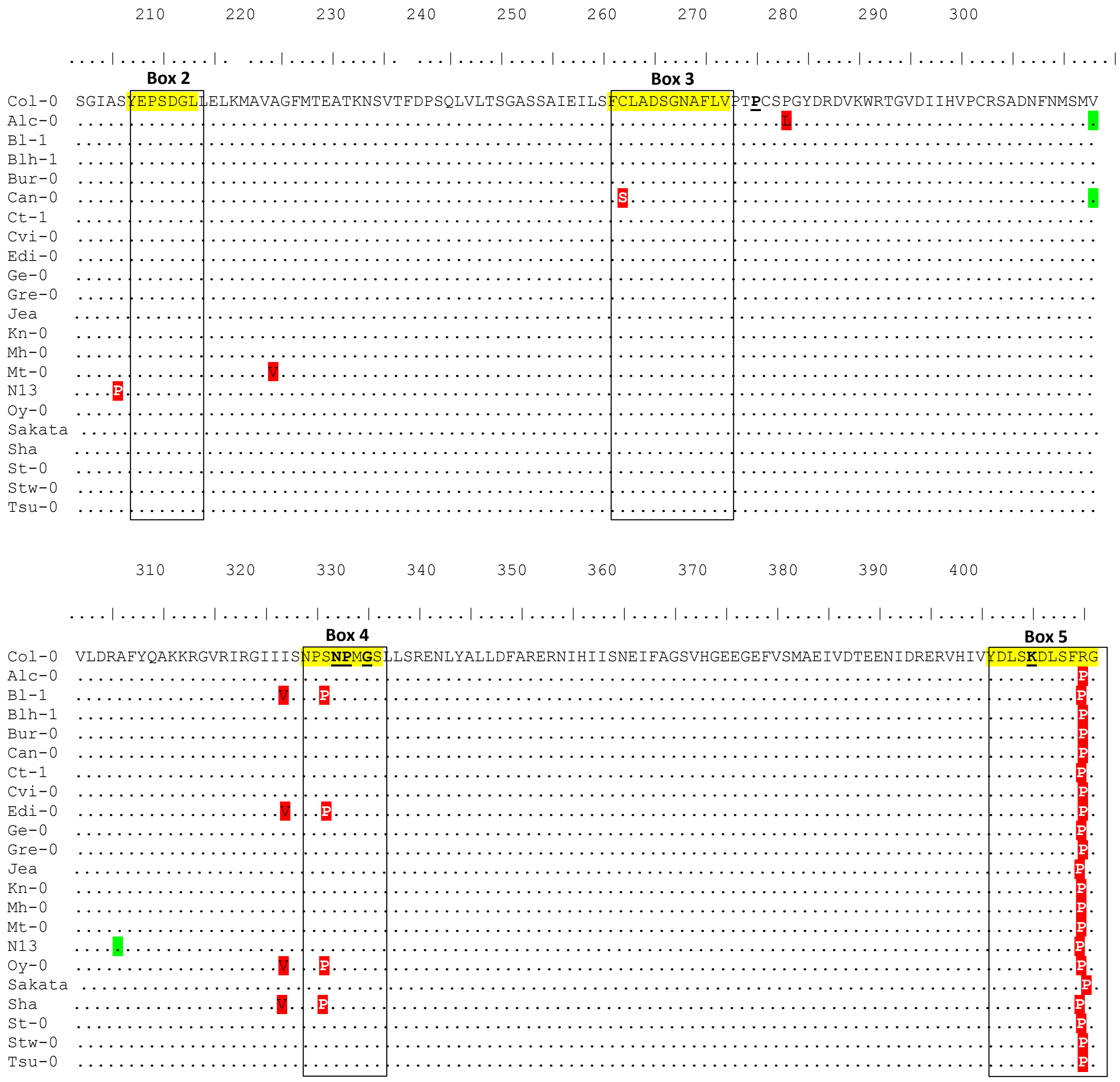

210 220 230 240 250 260 270 280 290 300
Box 2
Box 3
Col-0 SGIASYEPSDGLLELKMAVAGFMTEATKNSVTFDPSQLVLTSGASSAIEILSFCLADSGNAFLVPTPCSPGYDRDVKWRTGVDIIHVPCRSADNFNMSMV
Alc-0 L
Bl-1
Blh-1
Bur-0
Can-0 S
Ct-1
Cvi-0
Edi-0
Ge-0
Gre-0
Jea
Kn-0
Mh-0
Mt-0 V
N13 P
Oy-0
Sakata
Sha
St-0
Stw-0
Tsu-0
310 320 330 340 350 360 370 380 390 400
Box 4
Box 5
Col-0 VLDRAFYQAKKRGVRIRGIIISNPSNPMGSLLSRENLYALLDFARERNIHIISNEIFAGSVHGEEGEFVSMAEIVDTEENIDRERVHIVYDLSKDLSFRG
Alc-0 P
Bl-1 V P P
Blh-1 P
Bur-0 P
Can-0 P
Ct-1 P
Cvi-0 P
Edi-0 V P P
Ge-0 P
Gre-0 P
Jea P
Kn-0 P
Mh-0 P
Mt-0 P
N13 P
Oy-0 V P P
Sakata P
Sha V P P
St-0 P
Stw-0 P
Tsu-0 P

```
         410       420       430       440       450       460       470       480       490       500
       Box 5                Box 6
Col-0  LRSAAIYSFNESVLSASRKLTTLSPVSSPTQHLLISAISNPKNVQRFVKTNRQRLQSIYTELVEGLKELGIECTRSNGGFYCWADMRGLISSYSEKGEIE
Alc-0  ....................................................................................................
Bl-1   .................K.....................T............................................................
Blh-1  ....................................................................................................
Bur-0  ....................................................................................................
Can-0  ....................................................................................................
Ct-1   ........................................................................................F...........
Cvi-0  ....................................................................................................
Edi-0  .................K.....................T............................................................
Ge-0   ....................................................................................................
Gre-0  ....................................................................................................
Jea    ....................................................................................................
Kn-0   ....................................................................................................
Mh-0   ....................................................................................................
Mt-0   ....................................................................................................
N13    ...............................Q....................................................................
Oy-0   .................K.....................T............................................................
Sakata ....................................................................................................
Sha    .................K.....................T............................................................
St-0   ....................................................................................................
Stw-0  .........................................................................................F..........
Tsu-0  ....................................................................................................

         510       520       530       540       550
                             Box 7
Col-0  LWNKLLNIGKINVIPGSCCHCIEPGWFRICFSNLSERDVPVVMNRIRKVCETCKSQNX
Alc-0  ..........................................................
Bl-1   ..........................................................
Blh-1  ..........................................................
Bur-0  ..........................................................
Can-0  ..........................................................
Ct-1   ..........................................................
Cvi-0  ..........................................................
Edi-0  ..........................................................
Ge-0   ..........................................................
Gre-0  ..........................................................
Jea    ..........................................................
Kn-0   ..........................................................
Mh-0   ..........................................................
Mt-0   ..........................................................
N13    ..........................................................
Oy-0   ..........................................................
Sakata ..........................................................
Sha    ..........................................................
St-0   ..........................................................
Stw-0  ..........................................................
Tsu-0  ..........................................................
```

ACS11

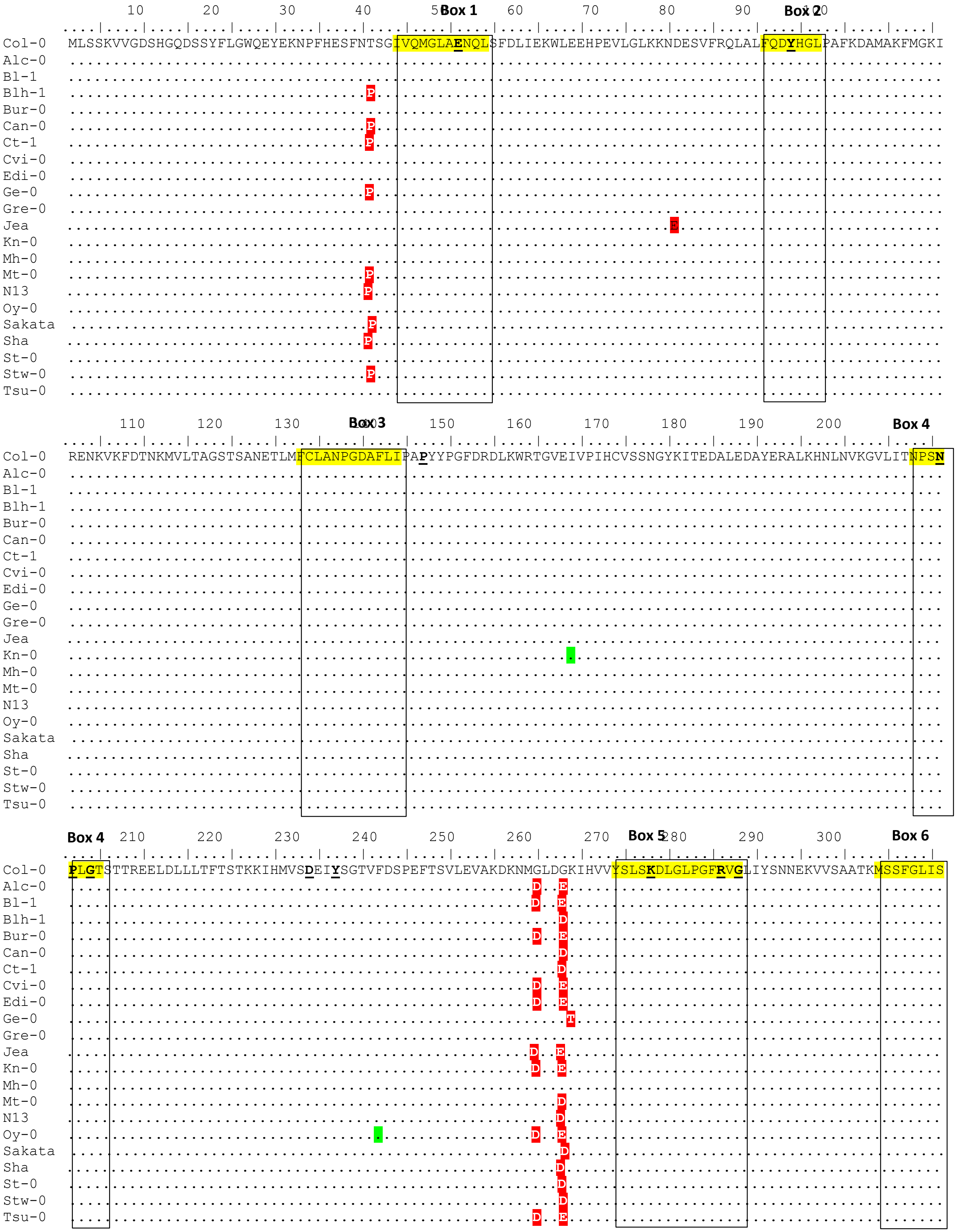
Box 1
Box 2
Box 3
Box 4
Box 5
Box 6
Col-0 MLSSKVVGDSHGQDSSYFLGWQEYEKNPFHESFNTSGIVQMGLAENQLSFDLIEKWLEEHPEVLGLKKNDESVFRQLALFQDYHGLPAFKDAMAKFMGKI
Col-0 RENKVKFDTNKMVLTAGSTSANETLMFCLANPGDAFLIPAPYYPGFDRDLKWRTGVEIVPIHCVSSNGYKITEDALEDAYERALKHNLNVKGVLITNPSN
Col-0 PLGTSTTREELDLLLTFTSTKKIHMVSDEIYSGTVFDSPEFTSVLEVAKDKNMGLDGKIHVVYSLSKDLGLPGFRVGLIYSNNEKVVSAATKMSSFGLIS
Alc-0
Bl-1
Blh-1
Bur-0
Can-0
Ct-1
Cvi-0
Edi-0
Ge-0
Gre-0
Jea
Kn-0
Mh-0
Mt-0
N13
Oy-0
Sakata
Sha
St-0
Stw-0
Tsu-0

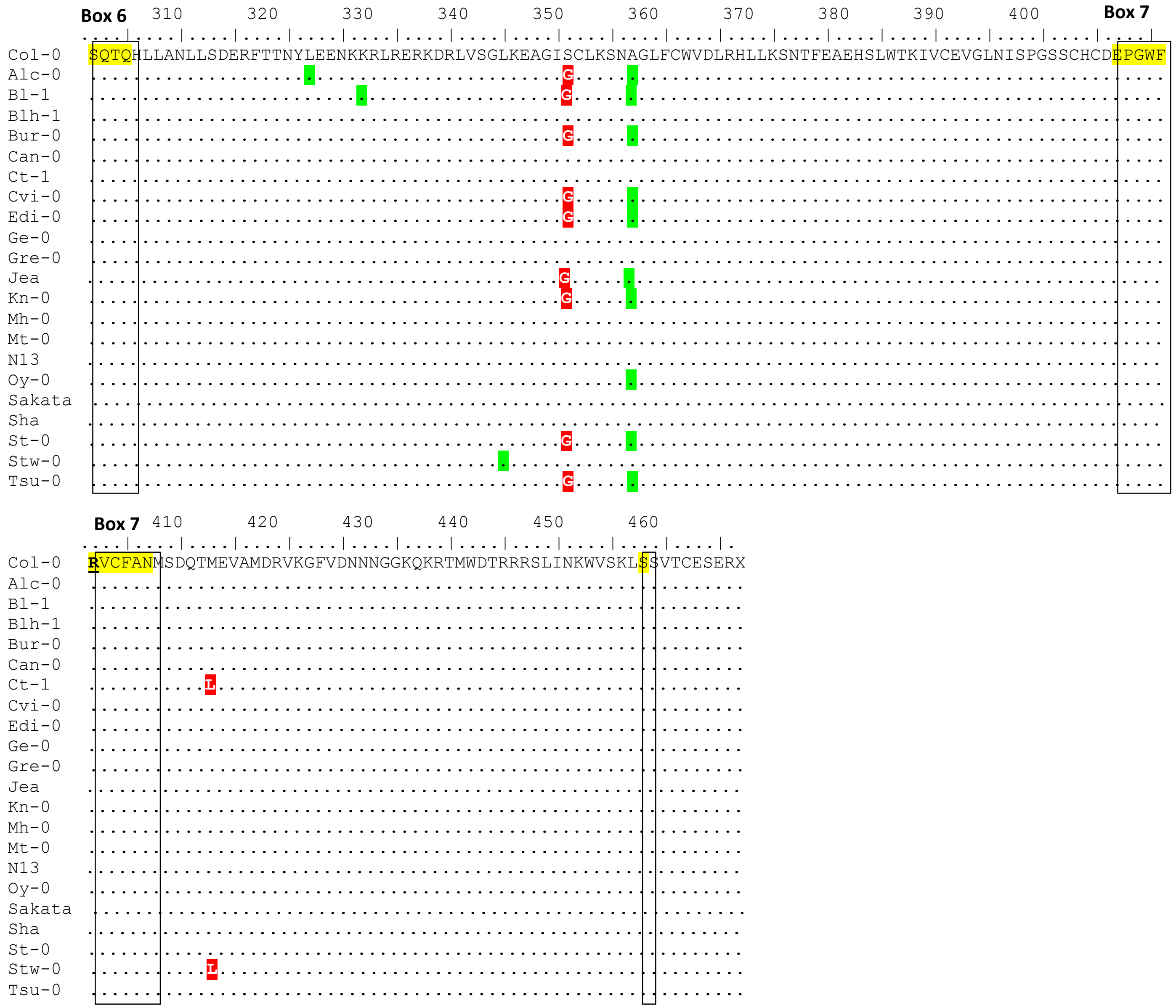
Box 6
310 320 330 340 350 360 370 380 390 400
Box 7
Col-0 SQTQHLLANLLSDERFTTNYLEENKKRLRERKDRLVSGLKEAGISCLKSNAGLFCWVDLRHLLKSNTFEAEHSLWTKIVCEVGLNISPGSSCHCDEPGWF
Alc-0
Bl-1
Blh-1
Bur-0
Can-0
Ct-1
Cvi-0
Edi-0
Ge-0
Gre-0
Jea
Kn-0
Mh-0
Mt-0
N13
Oy-0
Sakata
Sha
St-0
Stw-0
Tsu-0
G
Box 7
410 420 430 440 450 460
Col-0 RVCFANMSDQTMEVAMDRVKGFVDNNNGGKQKRTMWDTRRRSLINKWVSKLSSVTCESERX
L

ACS12

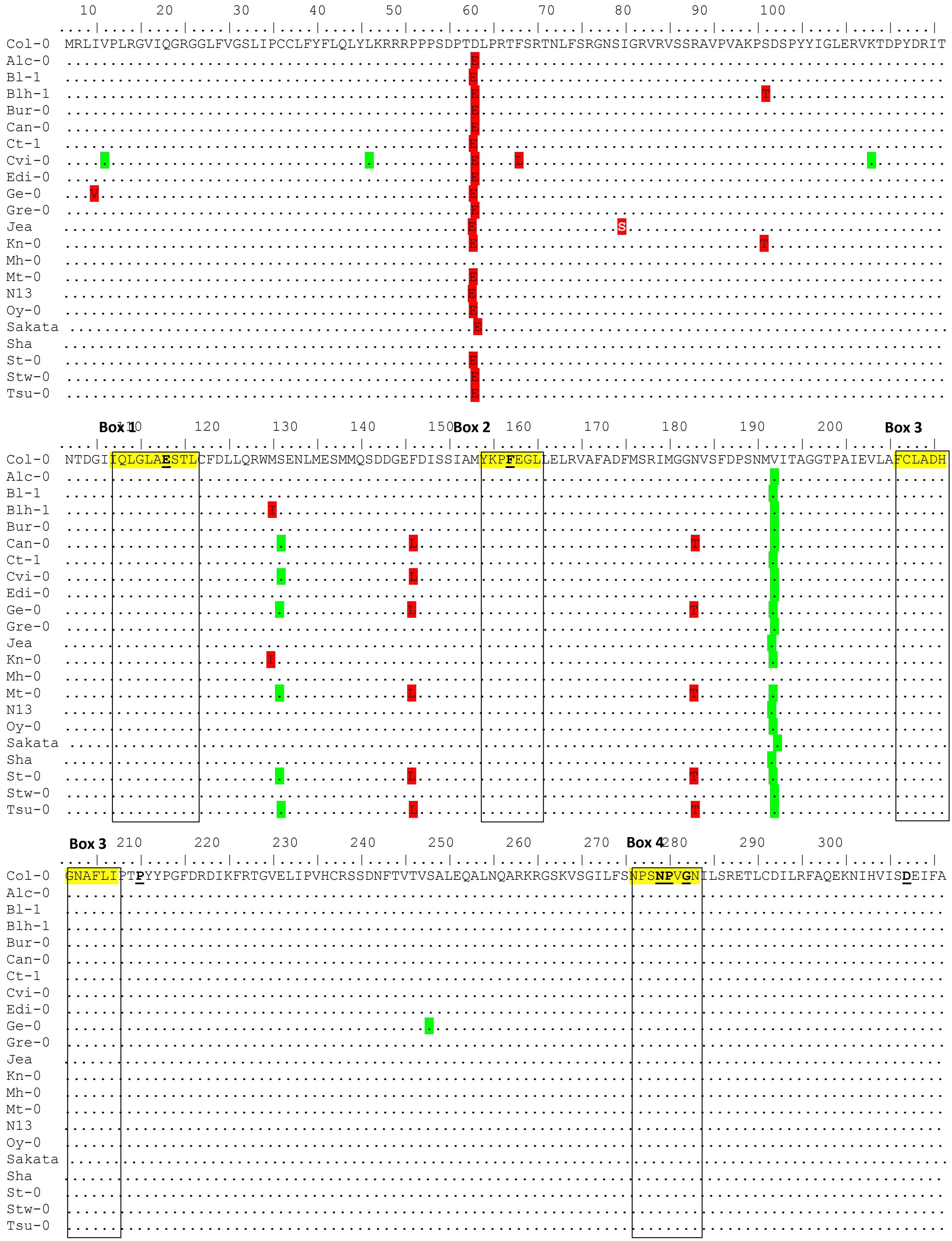

10 20 30 40 50 60 70 80 90 100
Col-0 MRLIVPLRGVIQGRGGLFVGSLIPCCLFYFLQLYLKRRRPPPSDPTDLPRTFSRTNLFSRGNSIGRVRVSSRAVPVAKPSDSPYYIGLERVKTDPYDRIT
Box 1 110 120 130 140 150 Box 2 160 170 180 190 200 Box 3
Col-0 NTDGIIQLGLAESTLCFDLLQRWMSENLMESMMQSDDGEFDISSIAMYKPFEGLLELRVAFADFMSRIMGGNVSFDPSNMVITAGGTPAIEVLAFCLADH
Box 3 210 220 230 240 250 260 270 Box 4 280 290 300
Col-0 GNAFLIPTPYYPGFDRDIKFRTGVELIPVHCRSSDNFTVTVSALEQALNQARKRGSKVSGILFSNPSNPVGNILSRETLCDILRFAQEKNIHVISDEIFA
Alc-0
Bl-1
Blh-1
Bur-0
Can-0
Ct-1
Cvi-0
Edi-0
Ge-0
Gre-0
Jea
Kn-0
Mh-0
Mt-0
N13
Oy-0
Sakata
Sha
St-0
Stw-0
Tsu-0

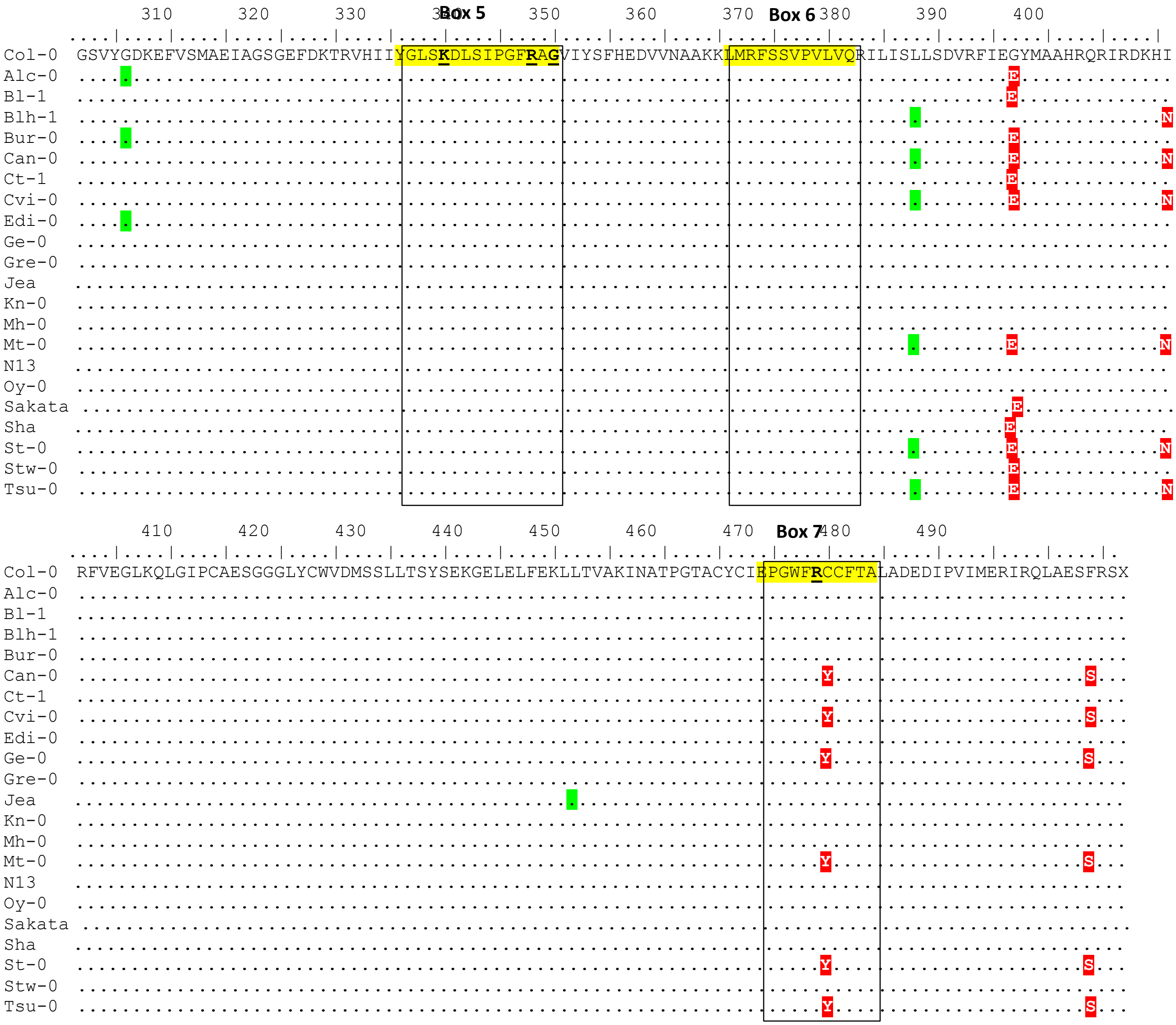

310 320 330 340 350 360 370 380 390 400
Box 5
Box 6
Col-0 GSVYGDKEFVSMAEIAGSGEFDKTRVHIIYGLSKDLSIPGFRAGVIYSFHEDVVNAAKKLMRFSSVPVLVQRILISLLSDVRFIEGYMAAHRQRIRDKHI
Alc-0
Bl-1
Blh-1
Bur-0
Can-0
Ct-1
Cvi-0
Edi-0
Ge-0
Gre-0
Jea
Kn-0
Mh-0
Mt-0
N13
Oy-0
Sakata
Sha
St-0
Stw-0
Tsu-0
410 420 430 440 450 460 470 480 490
Box 7
Col-0 RFVEGLKQLGIPCAESGGGLYCWVDMSSLLTSYSEKGELELFEKLLTVAKINATPGTACYCIEPGWFRCCFTALADEDIPVIMERIRQLAESFRSX
Alc-0
Bl-1
Blh-1
Bur-0
Can-0
Ct-1
Cvi-0
Edi-0
Ge-0
Gre-0
Jea
Kn-0
Mh-0
Mt-0
N13
Oy-0
Sakata
Sha
St-0
Stw-0
Tsu-0
