# Supplementary material for: A Comparative Study of Ethylene Emanation upon Nitrogen Deficiency in Natural Accessions of Arabidopsis thaliana
Source: Front Plant Sci. 2016 Feb 10;7:70. doi: 10.3389/fpls.2016.00070 (PMC4748056; doi:10.3389/fpls.2016.00070)
Supplement: Supplementary file 10 [file Image3.PDF]

Fig S3 | Amino acid sequence alignment of ACC oxidase proteins in Arabidopsis accessions.

The amino acid sequences of five ACO isozymes from 21 accessions were retrieved from Salk Arabidopsis 1,001 Genomes database and compared to Col-0 reference accession. Perfect matches are indicated by '.' and deletions by '/'. Synonymous substitutions are indicated in green. Non-synonymous substitutions are highlighted in red, with a black letter indicating a conservative substitution, which maintains the property of the amino acids (acidic polar: D, E; basic polar: H, K, R; neutral polar: N, Q, S, T, Y; neutral slightly polar: C, W; neutral non-polar: A, F, G, I, L, M, P, V) and with a white letter indicating a non-conservative substitution (change between those groups). The so-called '2-His-1-carboylate facial triad' (HxD…H) motif is involved in co-factor Fe(II) binding pocket, while the RxS motif is critical for co-substrate (ascorbate molecule) binding pocket. Further description on conserved motifs can be found in Seo et al. (2004) and Yuan et al. (2010).

ACO1

```
                 10        20        30        40        50        60        70        80        90        100
        ....|....|....|....|....|....|....|....|....|....|....|....|....|....|....|....|....|....|....|....|....|....|....|
Col-0   MVLIKEREMEIPVIDFAELDGEKRSKTMSLLDHACDKWGFFMVDNHGIDKELMEKVKKMINSHYEEHLKEKFYQSEMVKALSEGKTSDADWESSFFISHK
Alc-0   ....................................................................................................
Bl-1    ....................................................................................................
Blh-1   ....................................................................................................
Bur-0   ....................................................................................................
Can-0   ....................................................................................................
Ct-1    ....................................................................................................
Cvi-0   ....................................................................................................
Edi-0   .//////////.........................................................................................
Ge-0    ....................................................................................................
Gre-0   ....................................................................................................
Jea     ....................................................................................................
Kn-0    ....................................................................................................
Mh-0    ....................................................................................................
Mt-0    .//////////.........................................................................................
N13     ....................................................................................................
Oy-0    ....................................................................................................
Sakata  ....................................................................................................
Sha     ....................................................................................................
St-0    ....................................................................................................
Stw-0   ....................................................................................................
Tsu-0   ....................................................................................................

                110       120       130       140       150       160       170       180       190       200
        ....|....|....|....|....|....|....|....|....|....|....|....|....|....|....|....|....|....|....|....|....|....|....|
Col-0   PTSNICQIPNISEELSKTMDEYVCQLHKFAERLSKLMCENLGLDQEDIMNAFSGPKGPAFGTKVAKYPECRPRPELMRGLREHTDAGGIILLLQDDQVPGL
Alc-0   ....................................................................................................
Bl-1    ....................................................................................................
Blh-1   ....................................................................................................
Bur-0   ....................................................................................................
Can-0   ....................................................................................................
Ct-1    ....................................................................................................
Cvi-0   ....................................................................................................
Edi-0   ....................................................................................................
Ge-0    ....................................................................................................
Gre-0   ....................................................................................................
Jea     ....................................................................................................
Kn-0    ....................................................................................................
Mh-0    ....................................................................................................
Mt-0    ....................................................................................................
N13     ....................................................................................................
Oy-0    ....................................................................................................
Sakata  ....................................................................................................
Sha     ....................................................................................................
St-0    ....................................................................................................
Stw-0   ....................................................................................................
Tsu-0   ....................................................................................................
```

```
                210       220       230       240       250       260       270       280       290       300
         ....|....|....|....|....|....|....|....|....|....|....|....|....|....|....|....|....|....|....|....|
Col-0    EFFKDGKWVPIPPSKNNTIFVNTGDQLEILSNGRYKSVVHRVMTVKHGSRLSIATFYNPAGDAIISPAPKLLYPSGYRFQDYLKLYSTTKFGDKGPRLET
Alc-0    ....................................................................................................
Bl-1     ....................................................................................................
Blh-1    ....................................................................................................
Bur-0    ...............K....................................................................................
Can-0    ...............................................................................................S....
Ct-1     ....................................................................................................
Cvi-0    ...............................................................................................S....
Edi-0    ....................................................................................................
Ge-0     ....................................................................................................
Gre-0    ....................................................................................................
Jea      ....................................................................................................
Kn-0     ....................................................................................................
Mh-0     ....................................................................................................
Mt-0     ....................................................................................................
N13      ....................................................................................................
Oy-0     ...............K....................................................................................
Sakata   ....................................................................................................
Sha      ....................................................................................................
St-0     ....................................................................................................
Stw-0    ....................................................................................................
Tsu-0    ....................................................................................................

                310
         ....|....|.
Col-0    MKKMGNADSAX
Alc-0    ...........
Bl-1     ...........
Blh-1    ...........
Bur-0    ...........
Can-0    ...........
Ct-1     ...........
Cvi-0    ...........
Edi-0    ...........
Ge-0     ...........
Gre-0    ...........
Jea      ...........
Kn-0     ...........
Mh-0     ...........
Mt-0     ...........
N13      ...........
Oy-0     ...........
Sakata   ...........
Sha      ...........
St-0     ...........
Stw-0    ...........
Tsu-0    ...........
```

ACO2

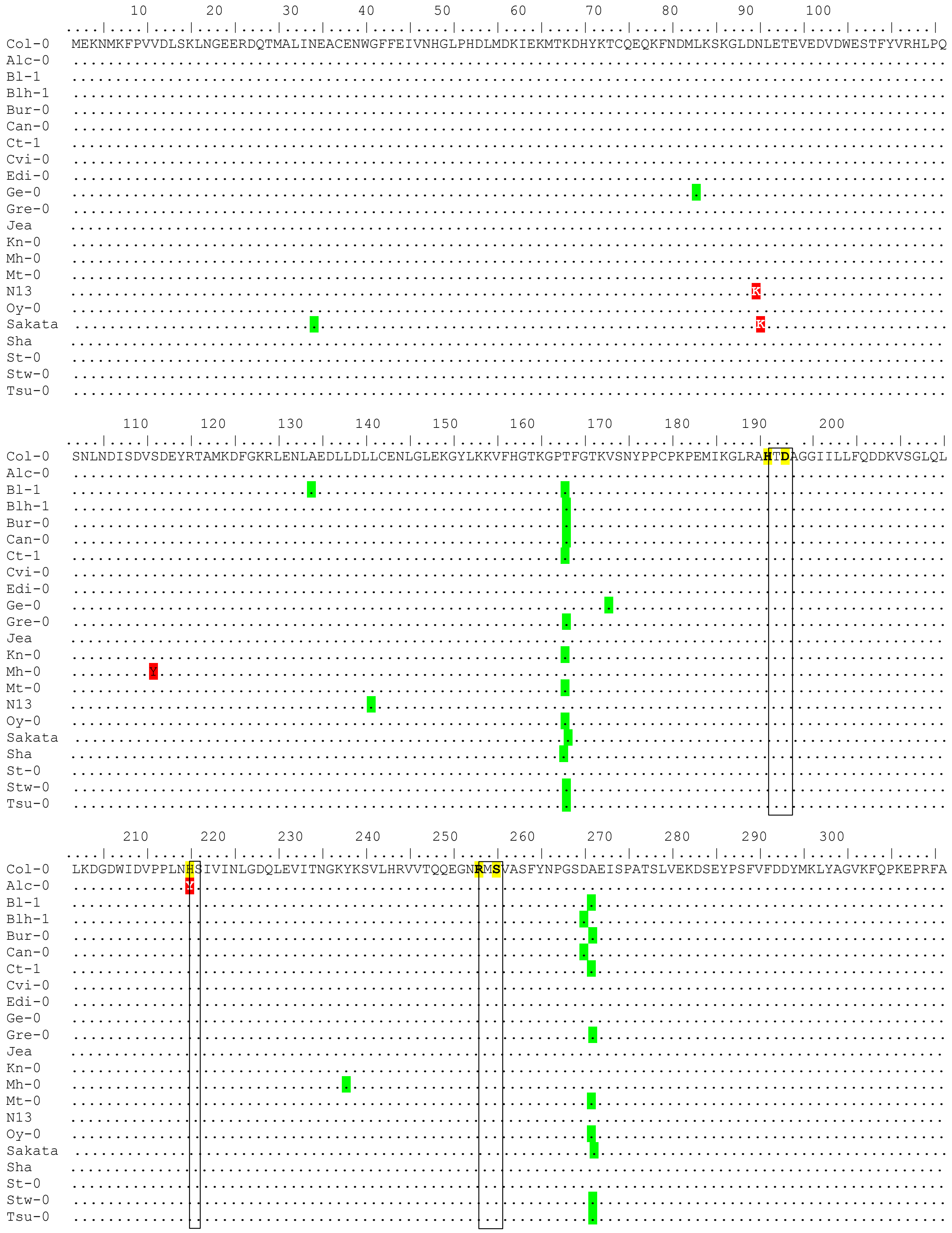

10 20 30 40 50 60 70 80 90 100
Col-0 MEKNMKFPVVDLSKLNGEERDQTMALINEACENWGFFEIVNHGLPHDLMDKIEKMTKDHYKTCQEQKFNDMLKSKGLDNLETEVEDVDWESTFYVRHLPQ
110 120 130 140 150 160 170 180 190 200
Col-0 SNLNDISDVSDEYRTAMKDFGKRLENLAEDLLDLLCENLGLEKGYLKKVFHGTKGPTFGTKVSNYPPCPKPEMIKGLRAHTDAGGIILLFQDDKVSGLQL
210 220 230 240 250 260 270 280 290 300
Col-0 LKDGDWIDVPPLNHSIVINLGDQLEVITNGKYKSVLHRVVTQQEGNRMSVASFYNPGSDAEISPATSLVEKDSEYPSFVFDDYMKLYAGVKFQPKEPRFA
Alc-0
Bl-1
Blh-1
Bur-0
Can-0
Ct-1
Cvi-0
Edi-0
Ge-0
Gre-0
Jea
Kn-0
Mh-0
Mt-0
N13
Oy-0
Sakata
Sha
St-0
Stw-0
Tsu-0
K
Y

```
                 310       320
          ....|....|....|....|.
Col-0     AMKNASAVTELNPTAAVETFX
Alc-0     .....................
Bl-1      ...........S.........
Blh-1     ...........S.........
Bur-0     ...........S.........
Can-0     ...........S.........
Ct-1      ...........S.........
Cvi-0     .....................
Edi-0     .....................
Ge-0      .....................
Gre-0     ...........S.........
Jea       ...........S.........
Kn-0      ...........S.........
Mh-0      .....................
Mt-0      ...........S.........
N13       .....................
Oy-0      ...........S.........
Sakata    ...........S.........
Sha       .....................
St-0      .....................
Stw-0     ...........S.........
Tsu-0     ...........S.........
```

ACO4

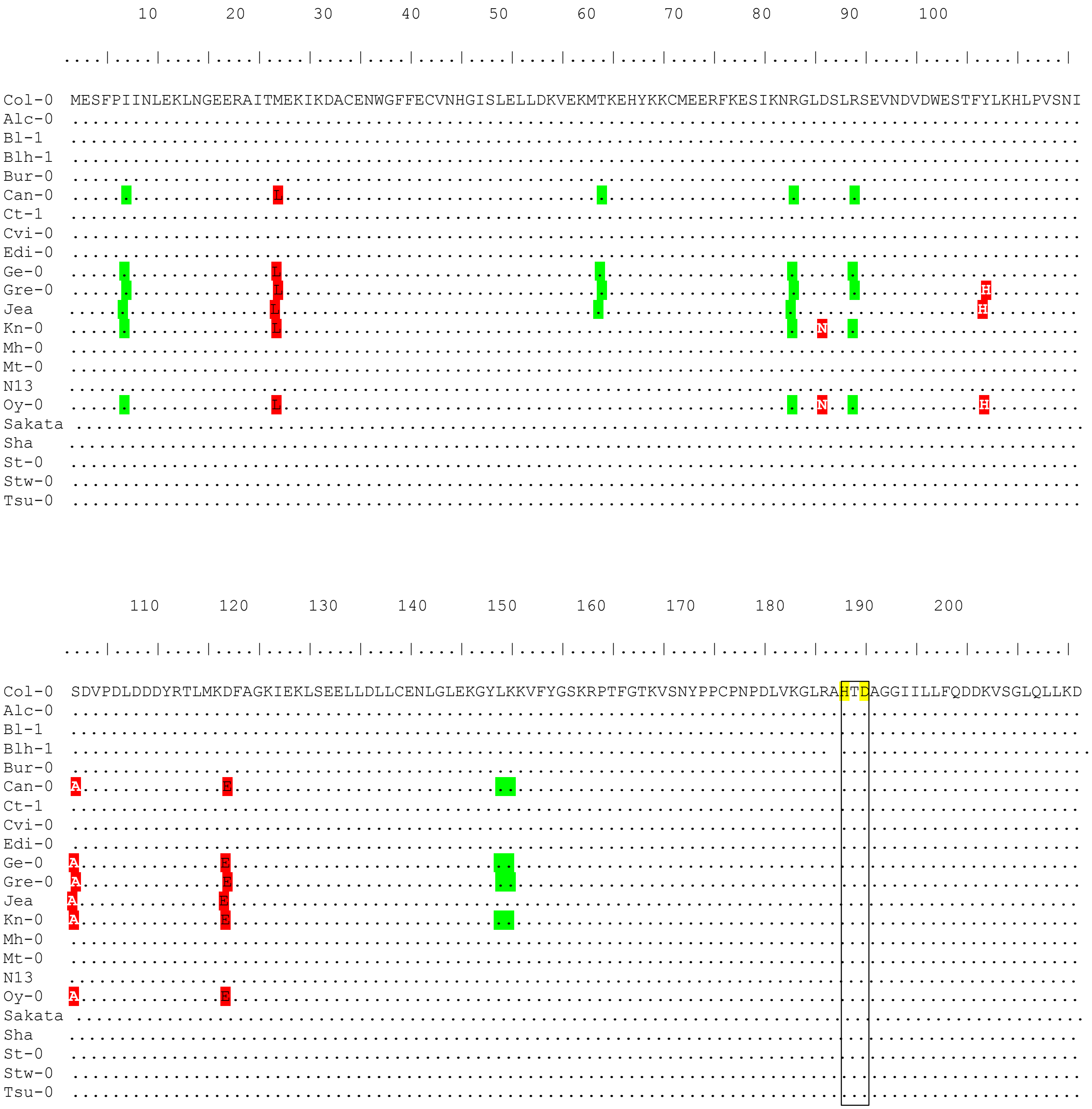
10 20 30 40 50 60 70 80 90 100
Col-0 MESFPIINLEKLNGEERAITMEKIKDACENWGFFECVNHGISLELLDKVEKMTKEHYKKCMEERFKESIKNRGLDSLRSEVNDVDWESTFYLKHLPVSNI
Alc-0
Bl-1
Blh-1
Bur-0
Can-0
Ct-1
Cvi-0
Edi-0
Ge-0
Gre-0
Jea
Kn-0
Mh-0
Mt-0
N13
Oy-0
Sakata
Sha
St-0
Stw-0
Tsu-0
110 120 130 140 150 160 170 180 190 200
Col-0 SDVPDLDDDYRTLMKDFAGKIEKLSEELLDLLCENLGLEKGYLKKVFYGSKRPTFGTKVSNYPPCPNPDLVKGLRAHTDAGGIILLFQDDKVSGLQLLKD

```
             210       220       230       240       250       260       270       280       290       300
         ....|....|....|....|....|....|....|....|....|....|....|....|....|....|....|....|....|....|....|....|
Col-0    GEWVDVPPVKHSIVVNLGDQLEVITNGKYKSVEHRVLSQTDGEGRMSIASFYNPGSDSVIFPAPELIGKEAEKEKKENYPRFVFEDYMKLYSAVKFQAKE
Alc-0    ....................................................................................................
Bl-1     .......................................... .........................................................
Blh-1    ....................................................................................................
Bur-0    ....................................................................................................
Can-0    ....................................................................................................
Ct-1     ....................................................................................................
Cvi-0    ....................................................................................................
Edi-0    ....................................................................................................
Ge-0     ....................................................................................................
Gre-0    ....................................................................................................
Jea      ....................................................................................................
Kn-0     ....................................................................................................
Mh-0     ....................................................................................................
Mt-0     ....................................................................................................
N13      ....................................................................................................
Oy-0     ....................................................................................................
Sakata   ....................................................................................................
Sha      ....................................................................................................
St-0     ....................................................................................................
Stw-0    ....................................................................................................
Tsu-0    ....................................................................................................

             310       320
         ....|....|....|....|....
Col-0    PRFEAMKAMETTVANNVGPLATAX
Alc-0    ........................
Bl-1     ........................
Blh-1    ........................
Bur-0    ........................
Can-0    ........................
Ct-1     ........................
Cvi-0    ........................
Edi-0    ........................
Ge-0     ........................
Gre-0    ........................
Jea      ........................
Kn-0     ........................
Mh-0     ........................
Mt-0     ........................
N13      ........................
Oy-0     ........................
Sakata   ........................
Sha      ........................
St-0     ........................
Stw-0    ........................
Tsu-0    ........................
```

ACO At1g12010

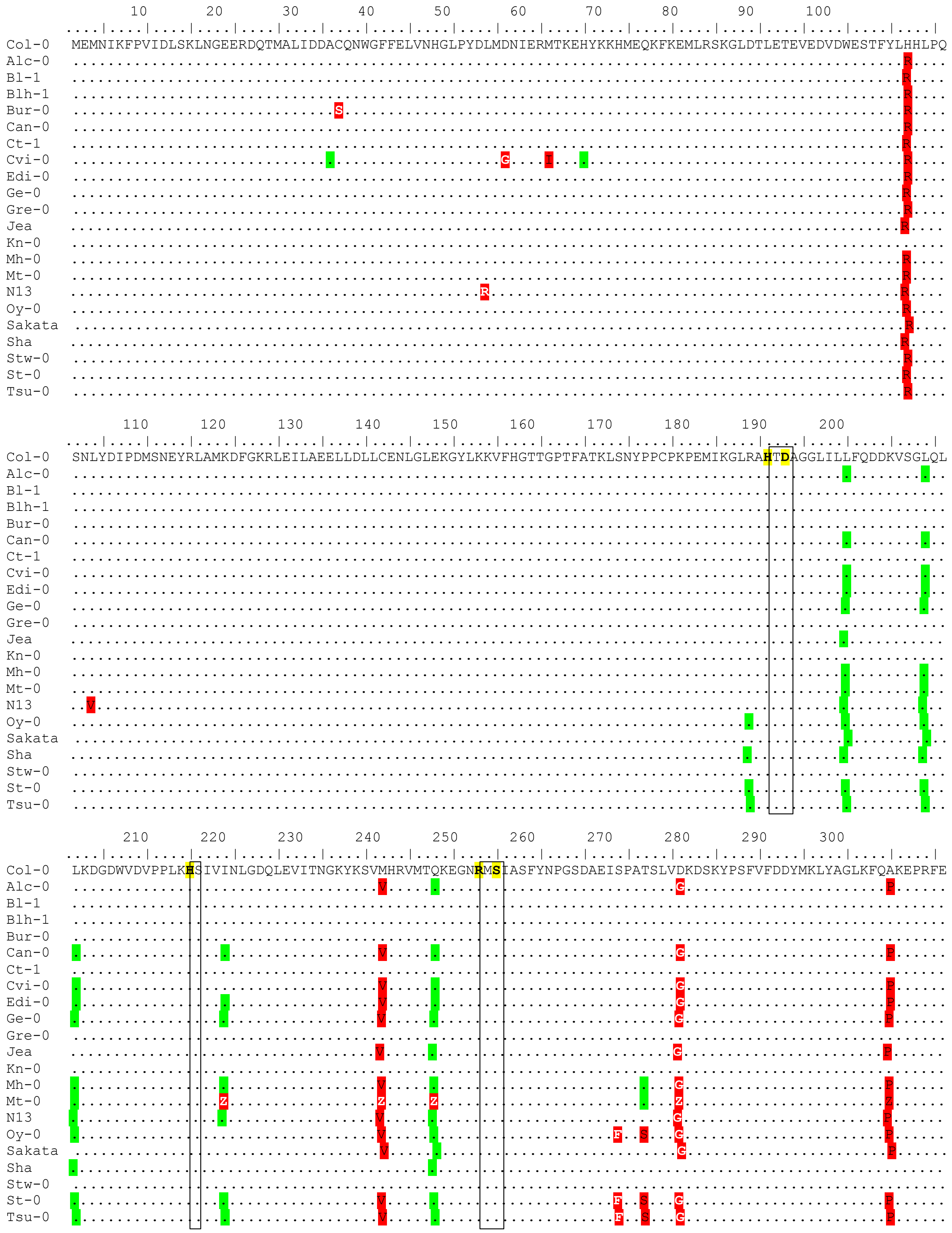

```
              310       320
         ....|....|....|....|.
Col-0    AMKNAEAAADLNPVAVVETFX
Alc-0    .....................
Bl-1     .....................
Blh-1    .....................
Bur-0    .....................
Can-0    .......V.......A.....
Ct-1     .....................
Cvi-0    ...............A.....
Edi-0    ...............A.....
Ge-0     ...............A.....
Gre-0    .....................
Jea      .....................
Kn-0     .....................
Mh-0     .....................
Mt-0     ..Z..................
N13      ...............A.....
Oy-0     ...............A.....
Sakata   ..........///////////
Sha      ...//////////////////
Stw-0    .....................
St-0     ...............A.....
Tsu-0    ...............A.....
```

ACO At1g77330

```
                 10        20        30        40        50        60        70        80        90       100
        ....|....|....|....|....|....|....|....|....|....|....|....|....|....|....|....|....|....|....|....|
Col-0   MAIPVIDFSKLNGEEREKTLSEIARACEEWGFFQLVNHGIPLELLNKVKKLSSDCYKTEREEAFKTSNPVKLLNELVQKNSGEKLENVDWEDVFTLLDHN
Alc-0   ....................................................................................................
Bl-1    ....................................................................................................
Blh-1   ....................................................................................................
Bur-0   ....................................................................................................
Can-0   ....................................................................................................
Ct-1    .....................................................V..............................................
Cvi-0   ....................................................................................................
Edi-0   ....................................................................................................
Ge-0    ....................................................................................................
Gre-0   ....................................................................................................
Jea     .....................................................V..............................................
Kn-0    ....................................................................................................
Mh-0    ....................................................................................................
Mt-0    ....................................................................................................
N13     ....................................................................................................
Oy-0    ....................................................................................................
Sakata  ....................................................................................................
Sha     ....................................................................................................
St-0    ....................................................................................................
Stw-0   ....................................................................................................
Tsu-0   ....................................................................................................

                110       120       130       140       150       160       170       180       190       200
        ....|....|....|....|....|....|....|....|....|....|....|....|....|....|....|....|....|....|....|....|
Col-0   QNEWPSNIKETMGEYREEVRKLASKMMEVMDENLGLPKGYIKKAFNEGMEDGEETAFFGTKVSHYPPCPHPELVNGLRAHTDAGGVVLLFQDDEYDGLQV
Alc-0   ....................................................................................................
Bl-1    ....................................................................................................
Blh-1   ....................................................................................................
Bur-0   ....................................................................................................
Can-0   .............V......................................................................................
Ct-1    ....................................................................................................
Cvi-0   .............V......................................................................................
Edi-0   ....................................................................................................
Ge-0    ....................................................................................................
Gre-0   ....................................................................................................
Jea     .............V......................................................................................
Kn-0    ....................................................................................................
Mh-0    .............V......................................................................................
Mt-0    ....................................................................................................
N13     ....................................................................................................
Oy-0    ....................................................................................................
Sakata  .............V......................................................................................
Sha     ....................................................................................................
St-0    ....................................................................................................
Stw-0   ....................................................................................................
Tsu-0   ....................................................................................................
```

```
                210       220       230       240       250       260       270       280       290       300
       ....|....|....|....|....|....|....|....|....|....|....|....|....|....|....|....|....|....|....|....|
Col-0  LKDGEWIDVQPLPNAIVINTGDQIEVLSNGRYKSAWHRVLAREEGNRRSIASFYNPSYKAAIGPAAVAEEEGSEKKYPKFVFGDYMDVYANQKFMPKEPR
Alc-0  ....................................................................................................
Bl-1   ....................................................................................................
Blh-1  ....................................................................................................
Bur-0  ....................................................................................................
Can-0  ....................................................................................................
Ct-1   ....................................................................................................
Cvi-0  .............................................................................S......................
Edi-0  ....................................................................................................
Ge-0   ....................................................................................................
Gre-0  ....................................................................................................
Jea    ...S................................................................................................
Kn-0   ....................................................................................................
Mh-0   ....................................................................................................
Mt-0   ....................................................................................................
N13    ....................................................................................................
Oy-0   ....................................................................................................
Sakata ....................................................................................................
Sha    ....................................................................................................
St-0   ....................................................................................................
Stw-0  ....................................................................................................
Tsu-0  ....................................................................................................

       ....|...
Col-0  FLAVKSLX
Alc-0  ........
Bl-1   ........
Blh-1  ........
Bur-0  ........
Can-0  ........
Ct-1   ........
Cvi-0  ........
Edi-0  ........
Ge-0   ........
Gre-0  ........
Jea    ........
Kn-0   ........
Mh-0   ........
Mt-0   ........
N13    ........
Oy-0   ........
Sakata ........
Sha    ........
St-0   ........
Stw-0  ........
Tsu-0  ........
```
